# Emerging Synthesis Strategies of High-Entropy Intermetallic Nanocatalysts

**DOI:** 10.3390/nano16080472

**Published:** 2026-04-16

**Authors:** Jitao Lu, Weiying Yang, Jun Chen, Maiyong Zhu, Quan Zhang

**Affiliations:** 1 State Key Laboratory of Advanced Fiber Materials, College of Materials Science and Engineering, Donghua University, Shanghai 201620, China; 2230688@mail.dhu.edu.cn (J.L.); yangweiying@mail.dhu.edu.cn (W.Y.); 2230463@mail.dhu.edu.cn (J.C.); 2 School of Materials Science & Engineering, Jiangsu University, Zhenjiang 212013, China; 3 Dishui Lake Laboratory, Shanghai Dianji University, Shanghai 201306, China; 4 State Key Laboratory of Structural Chemistry, Fujian Institute of Research on the Structure of Matter, Chinese Academy of Sciences (CAS), Fuzhou 350002, China

**Keywords:** intermetallic, high-entropy alloy, synthesis, nanocatalysts

## Abstract

High-entropy intermetallic (HEI) nanomaterials have recently been established as a representative class of high-performance nanocatalysts. However, the synthesis of HEI nanomaterials remains a significant challenge, with only a limited number of types being reported to date. This review aims to provide a systematical overview of the current state of research on HEI nanomaterials. The synthesis strategies are first discussed, with a special emphasis on the pivotal role that supports play in the formation of HEI nanomaterials. Following this, we provide a summary of key catalytic applications and focus on the structure–performance relationships. Finally, the review concludes by discussing the challenges faced in this area and suggesting potential research directions for the future.

## 1. Introduction

**Figure 1:**
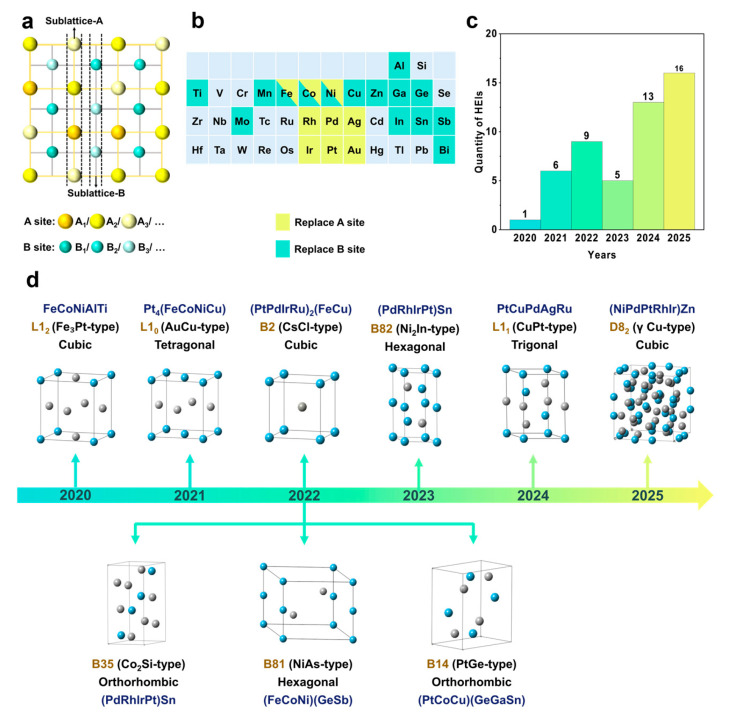
(**a**) Lattice structure model of HEIs, (**b**) elemental compositions of reported HEIs, (**c**) number histogram of HEIs, (**d**) timeline summarizing key developments in the synthesis of HEIs.

High-entropy intermetallic (HEI) nanomaterials have recently emerged as a promising class of nanocatalysts, demonstrating superior catalytic performance in several pivotal reactions relevant to the energy and chemical industries compared to conventional catalytic materials [1,2]. The concept of HEIs was introduced by Tsai in 2016 [3]. They defined that the HEIs have a crystal structure similar to binary intermetallics, but incorporate multiple principal elements like high-entropy alloys (HEAs), generally approaching an equimolar composition [3,4]. Currently, it is well established that HEIs form long-range ordered crystal structures, wherein specific crystallographic sites are preferentially occupied by distinct elements: A-site elements (e.g., A_1_/A_2_/…) occupy one sublattice, while B-site elements (e.g., B_1_/B_2_/B_3_/…) occupy another, thereby resulting in ordered sublattices. Within each sublattice, however, atomic occupancy remains random: A_1_ and A_2_ atoms are randomly distributed over the A-sites, while B_1_, B_2_, and B_3_ atoms randomly occupy the B-sites (Figure 1a) [5]. The distinctive atomic configuration of HEIs offers a synergistic combination of the benefits of HEAs and intermetallics. For instance, HEI nanoparticles exhibit abundant active sites due to their multi-element composition, thus highly promising for catalyzing complex multi-step reactions [6]. The ordered atomic arrangement facilitates site isolation, which can lower the homoatomic coordination number of active sites and provides exceptional selectivity for specific reaction pathways [7,8,9].

HEIs also possess exceptional thermodynamic stability due to their unique multicomposition and ordered atomic arrangement structure, leading to the outstanding catalytic stability. The multicompositional nature of HEIs resembles that of HEAs, as they share fundamental characteristics associated with HEAs, including the high-entropy effect, pronounced lattice distortion, and sluggish diffusion kinetics [10,11,12,13,14,15,16]. Moreover, the intermetallic structure of HEIs can effectively suppress atomic migration even under harsh working conditions, thereby exhibiting enhanced structural stability compared to HEA [17]. Regarding the benefits from the unique properties mentioned above, HEIs have shown remarkable efficacy and catalytic stability in numerous pivotal catalytic applications. Key examples encompass the propane dehydrogenation, alkyne semi-hydrogenation, formic acid oxidation reaction (FAOR), alcohol oxidation reaction (AOR), nitrate reduction reaction, hydrogen evolution reaction (HER) as well as the oxygen reduction reaction (ORR).

Theoretically, the vast compositional space of HEIs promises a broad variety of new materials. However, the development of HEIs is still in its infancy, with only 50 compounds across nine crystal structures (L1_0_, L1_1_, L1_2_, B2, B14, B35, B8_1_, B8_2_ and D8_2_) reported to date (Figure 1c,d). Among these HEIs, A sites are predominantly occupied by noble metals such as Pt, Rh, Pd, Ag, and Au. In some specific cases, Fe, Co, and Ni can also occupy the A sites. In contrast, B sites are typically filled by transition metals like Mn, Fe, Co, Ni, Cu and Zn, or liquid metals such as Ge, Ga, In, Sn, and Sb. To date, numerous approaches have been established for synthesizing HEIs (Figure 1b), with the majority showing a strong dependence on support materials. For example, the (PtPdIrRu)_2_(FeCu) [18], Pt_4_(FeCoNiCu) [19], (PtRh)(FeNiCu) [20] and (PtPdAu)(FeCo) [21] HEI nanomaterials were synthesized with the assistance of carbon black, sulfur-doped carbon, multiwalled carbon nanotubes and carbonized wood respectively. The synthesis of the (NiFeCu)(GaGe) [22], (PtCoCu)(GeGaSn) [23], (PtCoNi)(SnInGa) [24] and (PtIr)(FeMoBi) [25] HEIs was achieved by space-constrained annealing with the assistance of porous SiO_2_, porous CeO_2_ and polymer substrate-derived porous carbon. The Pt-Cu template was used to obtain the (PtPdAgRu)Cu and (PtPdAg)(CuFe) HEI nanomaterials [26]. Therefore, the carriers must play an important role during the synthesis of the HEIs (Table 1). However, owing to the complex synthesis conditions, a systematic understanding of the preparation methods for HEIs remains lacking. This knowledge gap, in turn, confines most current methods to a limited set of systems, hindering their broader preparation across different compound systems. Consequently, a systematic elucidation of the synthesis strategies and their governing mechanisms is paramount to accelerating the development of novel HEIs.

Several reviews on HEIs have already provided comprehensive discussions on their fundamental properties, synthetic pathways, and electrocatalytic applications [3,17,43]. This review specifically emphasizes emerging synthesis strategies for HEIs and tries to elucidate the mechanistic principles underlying these approaches. We aim to offer new insights that may inspire future developments in HEI synthesis. The review begins with an in-depth discussion of support-assisted synthesis, emphasizing how different supports influence HEI nucleation, growth, and structural evolution. Subsequent sections outline the latest advances in catalytic applications. Finally, we provide an outlook on future opportunities and challenges in HEI synthesis.

## 2. Synthesis Strategies

A systematic review of the reports on the preparation of HEI nanomaterials reveals that support materials play an important role in most of the syntheses. To better understand the influence of supports on the preparation of HEI nanomaterials, we therefore classify the synthesis methods based on the roles of supports, primarily including carrier-supported annealing synthesis and space-constrained annealing synthesis. Subsequently, we introduce a support-free liquid-phase synthesis approach.

### 2.1. Carrier-Supported Annealing

**Figure 2:**
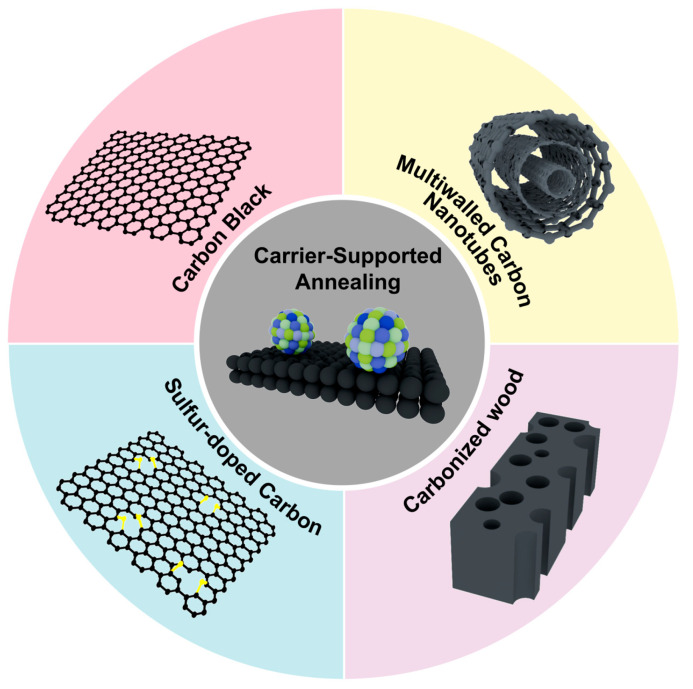
Materials for carrier-supported annealing synthesis of HEIs.

According to theoretical study, HEIs with ordered atomic arrangement usually possess high thermodynamic stability compared to disordered alloy structures. Therefore, annealing a disordered alloy at high temperature is the most popular way to obtain the ordered HEI, during which the disordered alloy will experience a disorder-to-order structural transition [44,45]. However, the high temperature and long heating time always induce sintering and aggregation of the nanocrystal. To obtain HEI catalysts with a desired particle size, carriers are usually used during the heat treatment process to prevent the aggregation [17,43,44,45,46,47]. Carbon-based materials with excellent thermostability, electrical conductivity and a large surface area have been widely used as carriers to support HEI nanomaterials because they can easily transfer the electrons and prevent particle aggregation, which are significant for catalytic process [48,49,50]. At present, four different types of carbon-based materials have been reported: carbon black, sulfur-doped carbon, multi-walled carbon nanotubes, and carbonized wood structures (Figure 2).

#### 2.1.1. Carbon Black-Assisted Annealing

Carbon black is an amorphous carbon material composed of nanoparticles, which is the most popular HEI carrier because of its unique physicochemical properties, including a large specific surface area, good stability, excellent electrical conductivity, tunable morphology and structure, relatively low cost, etc. [51,52]. The large specific surface area provides abundant sites for the high dispersion of nanoparticles, promoting the formation of small and uniformly distributed HEI nanoparticles. As a result, the loading amount of the metal catalysts has been reported to be as high as 392 wt% of the carbon black support, which will expose a large number of catalytic active sites and facilitate the overall catalytic process [53]. Carbon black also demonstrates a relatively high structural stability under high temperature, which enables it to maintain integrity under various harsh operational conditions [54]. Furthermore, its high chemical stability in both acidic and alkaline environments shows strong resistance to corrosion [55]. The excellent electrical conductivity of carbon black facilitates efficient charge transfer between active sites and the electrode, and it is thereby widely employed as a catalyst support in electrocatalysis. By selecting different synthesis methods and precursors, carbon black with tailored structures can be obtained, which offers opportunities to optimize the mass transfer of the reactants and products during the catalytic processes [56,57,58]. Compared to other types of carbon materials including carbon nanotubes and graphene, carbon black benefits from mature industrial production technologies, widely available raw materials, and low cost. These advantages make it economically favorable for large-scale commercial applications [59]. Currently, two types of commercial carbon black supports have been employed in the synthesis of HEIs. The first is Vulcan XC-72R (Cabot Corporation, USA), which exhibits a specific surface area of 250 m^2^/g and an electrical conductivity of 2.77 S/cm. The other, Ketjen EC-300J from Lion Specialty Chemicals, is a high-performance conductive carbon black with a much greater surface area (800 m^2^/g). This material requires only one-third of the loading of conventional conductive carbon black to attain equivalent electrical conductivity. Currently, the loading amount of the HEI catalysts has reached 40 wt% for both types of the carbon black support [27,31]. Moreover, these kinds of carbon can withstand temperatures up to 1200 °C [60].

**Figure 3:**
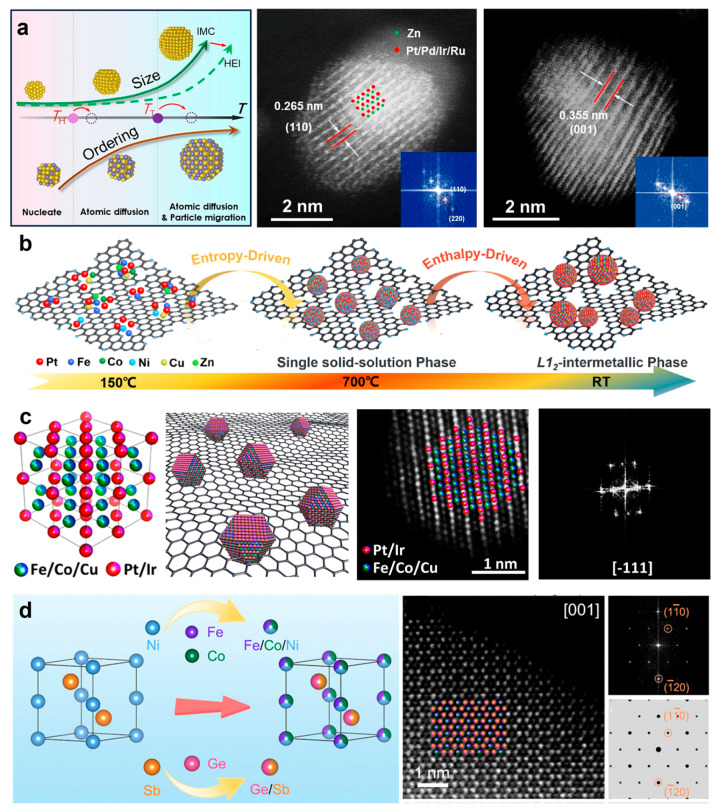
(**a**) Schematic diagram for the synthesis of (PtPdIrRu)_2_FeCu HEI nanoparticles. Reproduced with permission [18]. Copyright 2024, Wiley. (**b**) Schematic illustration for the preparation of Pt(FeCoNiCuZn)3/C. Reproduced with permission [27]. Copyright 2023, Elsevier. (**c**) Scheme of the evolution of PdFeCoNiCu HEI. Reproduced with permission [28]. Copyright 2024, American Chemical Society. (**d**) Schematic illustration of PtIrFeCoCu HEI NPs growing on the carbon supports. Reproduced with permission [30]. Copyright 2023, American Chemical Society. (**e**) Structure of FeCoNiGeSb HEI. Reproduced with permission [31]. Copyright 2025, Wiley.

Shen et al. synthesized a carbon-supported B2-(PtPdIrRu)_2_FeCu/C HEI through an impregnation-reduction method (Figure 3a) [18]. The Vulcan XC-72 carbon was uniformly dispersed into the metal precursor solution to form a slurry. This mixture was vacuum-dried and ground into a homogeneous powder. Then, the HEA nanoparticles were prepared by reducing the resulting powder in a H_2_/Ar atmosphere at 200 °C for 2 h. Finally, the (PtPdIrRu)_2_FeCu/C with an intermetallic structure was obtained by annealing the HEA nanoparticles at 500 °C for 2 h in the same atmosphere. Zhang et al. reported the synthesis of L1_2_-Pt(FeCoNiCuZn)_3_ nanoparticles with an acid-treated carbon black carrier (Figure 3b) [27]. Vulcan XC-72 carbon black was first dispersed in a concentrated nitric acid. Then, it was refluxed in an oil bath at 80 °C for 10 h. The Vulcan XC-72 treated by the acid was centrifuged and dried overnight for further use. The acid treatment can make metal salts uniformly disperse on the Vulcan XC-72 carbon black. H_2_PtCl_6_·6H_2_O, FeCl_3_·6H_2_O, Zn(NO_3_)_2_·6H_2_O, NiCl_2_·6H_2_O, CoCl_2_·6H_2_O, and CuCl_2_·2H_2_O were dissolved in deionized water dispersed with the acid-treated Vulcan XC-72. The mixture was stirred and heated until the solvent was totally evaporated. The obtained powder was dried overnight and reduced in H_2_/Ar (10%) at 150 °C for 2 h. Finally, the powder was annealed at 700 °C for another 2 h in the same atmosphere, and Pt(FeCoNiCuZn)_3_/C HEI is obtained. Liu et al. prepared B2-PdFeCoNiCu (PdM) HEI/C through phase transition of disorder to order under at a certain temperature (Figure 3c) [28]. Pd(acac)_2_, Ni(acac)_2_, Fe(acac)_3_, Co(acac)_3_, Cu(acac)_2_, and ascorbic acid were added to the mixture solution of oleic acid, oleylamine, and 1-octadecene and sonicated for 1 h with stirring. The solution was then heated to 220 °C and held for 2 h under magnetic stirring in an Ar atmosphere. Following that, the black colloidal products were collected and redispersed in hexane. Then, the redispersed colloid solution was mixed with VulcanXC-72R carbon in ethanol, sonicated for 20 min, and stirred overnight to form PdM/C powders. Finally, the PdFeCoNiCu/C nanoparticles were prepared by annealing the PdM/C powders at 500 °C under H_2_/Ar.

Nakamura et al. prepared the carbon black-supported B2-(PtRhRuPdIr)In HEI nanoparticles (In–PGM HEI NPs) through a two-step method [29]. First, HEA nanoparticles were synthesized through a wet-chemical reduction method. The metal precursors were dissolved in a mixture of tetraethylene glycol (TEG) and dibenzyl ether. Sodium borohydride (NaBH_4_) was dissolved in TEG as the reducing agent. The protective agent polyvinylpyrrolidone (PVP) was dissolved in TEG and heated to 200 °C under an Ar atmosphere. The metal precursor and reducing agent solutions were then co-injected into the preheated TEG solution at a rate of 1.0 mL/min. To compensate for NaBH_4_ decomposition at high temperatures, 5 mL of NaBH_4_ was added before injection, with an additional 5 mL added afterward. After the reduction, the reaction solution was quickly cooled in an ice-water bath, and the HEA nanoparticles were separated as a black powder by centrifugation. Second, the obtained HEA nanoparticles and Vulcan XC-72R were dispersed in ethanol together and sonicated for 1 h. The In–PGM HEI NPs/C were finally formed by heating in H_2_ at 350 °C for 20 min. Feng et al. synthesized the structurally ordered carbon-loaded L1_0_-PtIrFeCoCu HEI nanoparticles with Ketjen-Black as the carrier through a co-reduction and annealing method (Figure 3d) [30]. First, five metal salts (H_2_PtCl_6_·6H_2_O, IrCl_4_·nH_2_O, CuSO_4_·5H_2_O, FeSO_4_·7H_2_O, and CoSO_4_·7H_2_O) were dissolved in a weak acidic solution, followed by adding carbon black as the carrier with ultrasonic stirring. Then, NaBH_4_ was added to reduce the metal precursors. Finally, the PtIrFeCoCu HEI nanoparticles were obtained by annealing the separate resulting intermediate in H_2_/Ar at 850 °C for 4 h. Ma et al. synthesized B81-FeCoNiGeSb HEI NPs using a high-temperature annealing method in the presence of the Ketjen-Black EC300J (Figure 3e) [31]. First, the precursor salts, including Fe(NO_3_)_3_·9H_2_O, Co(NO_3_)_2_·6H_2_O, NiCl_2_·6H_2_O, C_8_H_4_K_2_O_12_Sb_2_·3H_2_O, and (NH_4_)_2_GeF_6_, were uniformly mixed with Ketjen-Black by grinding. The resulting mixture was then annealed in H_2_/Ar at 900 °C for 10 h to form the FeCoNiGeSb HEI nanoparticles.

#### 2.1.2. Sulfur-Doped Carbon-Assisted Annealing

Carbon materials, which show excellent electrical conductivity, a large surface area, and good chemical stability, are often viewed as ideal supports for electrocatalytic and multiphase catalytic applications. However, the chemical inertness of carbon surfaces, composed of *sp2* or *sp3* hybridized carbon atoms, generally leads to weak interactions with metals, which will induce metal aggregation during the catalyst process. Introducing heteroatoms into the carbon lattice or attaching functional groups to the surface is a promising way to substantially enhance their interactions with metal particles [61].

**Figure 4:**
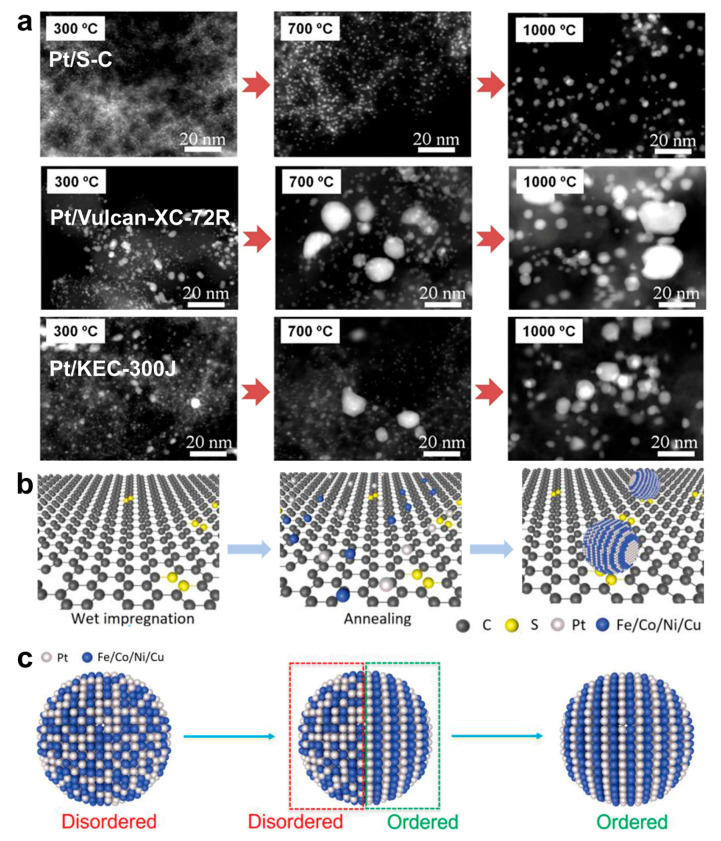
(**a**) The morphological changes to a Pt nanoparticle on different carbon supports with annealing. Reproduced with permission from [19]. Copyright 2021, AAAS. (**b**) Schematic illustration of Pt_4_FeCoCuNi nanoparticles synthesized through sulfur-doped carbon-assisted annealing. (**c**) Schematic illustration of the ordering of transformation for Pt_4_FeCoCuNi. Reproduced with permission [32]. Copyright 2023, Wiley.

Recently, Yang et al. reported porous sulfur-doped carbon supports, which were synthesized through a cobalt-assisted carbonation process with silica nanoparticles as templates. The sulfur-doped carbon showed a mesoporous structure with a large surface area of 1490 m^2^ g^−1^, which offers numerous uniformly distributed sulfur sites for anchoring metal nanoparticles [19]. The porous sulfur-doped carbon supports can induce strong metal–support interactions. Specifically, the strong bonding at the metal–sulfur interface significantly improves the adhesion between the carbon supports and the metals. Compared to carbon black, the sulfur-doped carbon showed greater resistance to Pt nanoparticle sintering, with Pt nanoparticles remaining under 1.5 nm even after annealing at 1000 °C (Figure 4a) [62]. Hence, 46 types of platinum-based alloys were synthesized on the sulfur-doped carbon support, including six kinds of HEI nanoparticles [19]. Taking the synthesis of Pt_4_FeCoCuNi HEI nanoparticles as an example, the porous sulfur-doped carbon was added into the precursor solutions with a Pt:Fe:Ni:Co atomic ratio of 4:1:1:1:1. The mixture was stirred overnight, and was dried with a rotary evaporator. Finally, the Pt_4_FeCoCuNi nanoparticles were obtained by annealing the dried sample in flowing Ar/H2 at 1000 °C for 2 h (Figure 4b,c) [32].

#### 2.1.3. Carbon Nanotube-Assisted Annealing

**Figure 5:**
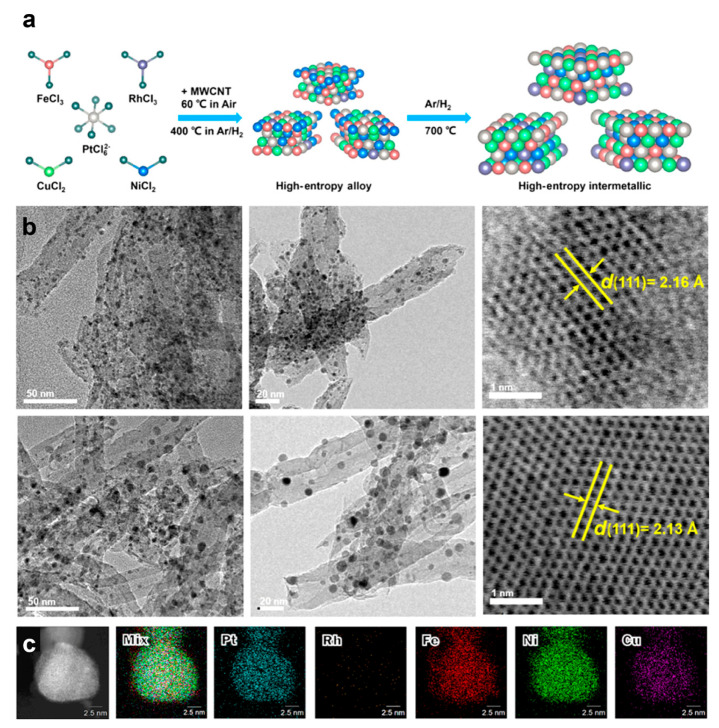
(**a**) Synthesis process of the PtRhFeNiCu HEI. (**b**) TEM images and high-resolution STEM image of the HEIs. (**c**) STEM-EDS elemental mapping of HEIs. Reproduced with permission from [20]. Copyright 2022, Wiley.

Multi-walled carbon nanotubes (MWCNTs), which are hollow, tubular structures composed of multiple concentric cylinders of graphene, are among the toughest known materials, exhibiting an extremely high Young’s modulus and tensile strength [63]. As a support, they can provide a robust framework that helps prevent the aggregation, sintering, or detachment of loaded nanoparticles during reactions, thereby extending the catalyst’s operational lifespan [64,65]. The high specific surface area (typically ranging from 150 to 1500 m^2^/g, depending on tube diameter, number of layers, and purity) provides abundant anchoring sites for loading functional nanoparticles, thereby effectively increasing the loading capacity and improving dispersion [66,67]. The MWCNTs also show exceptional electrical conductivity and mass transport due to their special graphene lattice structure, hollow inner cavities, inter-tube spaces, and one-dimensional nanowire-like structure [64,68]. When used as a support for electrocatalysts, MWCNTs can significantly facilitate electron transfer between the support and active sites, leading to markedly enhanced catalytic efficiency [69]. As a result, the MWCNTs were also investigated as carrier materials to synthesize HEI nanomaterials. Wang et al. described the synthesis of B2-PtRhFeNiCu HEI with MWCNTs as the carrier (Figure 5a–c) [20]. The MWCNTs were first functionalized by refluxing in a nitric and sulfuric acid mixture at 70 °C for 3 h. The functionalized MWCNTs were then added into the metal precursor solution, which dissolved them with H_2_PtCl_6_·6H_2_O, RhCl_3_·3H_2_O, CuCl_2_·2H_2_O, NiCl_2_·6H_2_O, and FeCl_3_. Next, the suspension was heated overnight with stirring until the water evaporated. The HEA precursor was prepared by grinding and heating the obtained powder at 60 °C for 12 h. Finally, the PtRhFeNiCu HEI was produced by annealing the HEA precursor in H_2_/Ar at 700 °C for 2 h.

#### 2.1.4. Carbonized Wood-Assisted Annealing

Carbonized wood was also used as the carrier for preparation of HEI nanoparticles, even though the detailed structure of carbonized wood was not clarified. Cui et al. reported the preparation of L1_0_-Pt(FeCoNiCu), L1_2_-(PtPdAu)(FeCoNiCuSn) and L1_2_-(PtPdAu)(FeCo) HEI nanoparticles using a Joule-heating method with carbonized wood as the carrier [21]. First, wood slices were continuously annealed at 260 °C in air and 1000 °C in Ar for 6 h, respectively. To create surface defects, the carbonized wood substrate was then treated in CO_2_ for 6 h at 750 °C. The precursor solution was mixed with carbonized wood and dried in an oven at 80 °C for 6 h. The HEA nanoparticles were synthesized with a Joule heating method, which was achieved by rapidly heating to ~827 °C for several tens of milliseconds. The synthesis of HEI nanoparticles was then driven through rapid reheating to ~827 °C for ~5 min. This step was precisely controlled to promote the desired transition to a single-phase HEI structure and simultaneously suppress the nanoparticle growth. Finally, the single-phase HEI nanoparticles with ~5 nm were prepared by rapid cooling at a rate of ~10^4^ K/s. It needs to be pointed out that the synthesis of HEA through rapid thermal shock can mix various elements without considering their thermodynamic miscibility. The following heating for approximately 5 min is crucial for transforming disordered HEA nanoparticles into HEI nanoparticles, which is driven by thermodynamic stability. Moreover, the short heating time and rapid cooling prevent excessive growth and agglomeration of HEI nanoparticles. The Joule-heating method, which allows precise control of heating by regulating electric current, not only produces stable HEIs at the desired nanoscale but also enables the creation of novel HEI compositions that are difficult to achieve with conventional methods due to the inherent immiscibility of the elements.

Despite the successful synthesis of many HEI nanomaterials via a carrier-assisted annealing method, its development faces a significant challenge. The reaction mechanism remains unclear due to its complex multiple coupled steps, such as precursor decomposition, reduction, diffusion, nucleation, and growth. Moreover, the influence of most supports in the formation of the HEI structure is largely unexplored, apart from sulfur-doped carbon. This lack of fundamental knowledge limits its scalable application to a broader range of HEI systems. Additionally, the high-temperature annealing process is inherently constrained by its enormous energy consumption and environmental concerns. Furthermore, the rational design of the support materials for HEI catalysts may greatly enhance their catalytic performance. For example, nitrogen-doped carbon materials, which are widely employed in fuel cell catalysis due to their strong metal–support interactions and good electronic conductivity, may represent a promising platform for stabilizing HEI nanostructures with improved catalytic activity.

### 2.2. Space-Constrained Annealing

**Figure 6:**
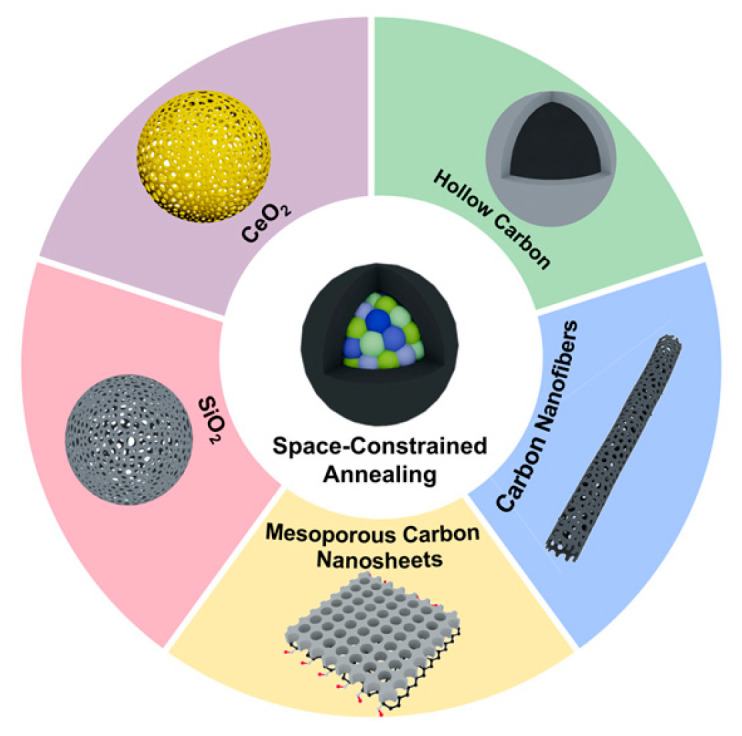
Substrates for space-constrained annealing.

Space-constrained annealing synthesis, which employs confined environments to guide atomic diffusion and ordering during the annealing process, is another important method to prepare HEI nanomaterials. The synthesis of long-range ordered intermetallic alloys needs long-range diffusion and reorganization of atoms. In high-entropy systems, this requirement is challenged by atomic size differences, known as the cocktail effect, which cause slow atomic diffusion rates and substantial kinetic barriers [12]. Traditional annealing strategies employing elevated temperatures and extended durations can overcome these barriers by supplying thermal energy; however, such treatments risk stabilizing a high-entropy state and potentially inducing the decomposition of the ordered phase or its transformation into a disordered solid solution structure [70,71,72]. Unlike the unrestricted atomic diffusion in traditional annealing synthesis, space-constrained annealing syntheses are conducted in nanoscale physically confined spaces [73,74,75]. During annealing, these confined spaces restrict atomic interdiffusion and ordering processes to highly localized regions, which suppresses the disordering tendencies in high-entropy systems. Consequently, it effectively overcomes the thermodynamic and kinetic barriers that typically inhibit the formation of ordered phases in high-entropy systems, thereby facilitating the directed synthesis of highly ordered intermetallic phases. Currently, five different space-constrained structures were employed to synthesize HEI nanomaterials, including SiO2, CeO2, hollow carbon, 1D carbon nanofibers and 2D mesoporous carbon nanosheets (Figure 6).

#### 2.2.1. Porous Silica-Constrained Annealing

Porous silica with pore channels ranged from 2 nm to 50 nm and a high melting point (approximately 1700 °C) is an important material for space-constrained annealing synthesis of nano HEI. Here, the small pore channels provide the space for the formation of HEI nanomaterials. During the high-temperature annealing process, the precursor species are physically isolated within the individual pores, in which the migration, aggregation, and sintering of the nanomaterials can be effectively suppressed, thereby enabling the formation of highly dispersed and uniformly sized nanoparticles. The high melting point allows it to withstand the elevated temperatures typically required for the synthesis and crystallization of HEI nanomaterials. In most cases, silica does not engage in side reactions with the confined precursors or the resulting nanomaterials, thereby ensuring high product purity. Currently, the commercial amorphous SiO_2_ named CARiACTG-6 and KIT-6 with a cubic structure are used for the synthesis of HEI nanoparticles [76,77,78,79].

**Figure 7:**
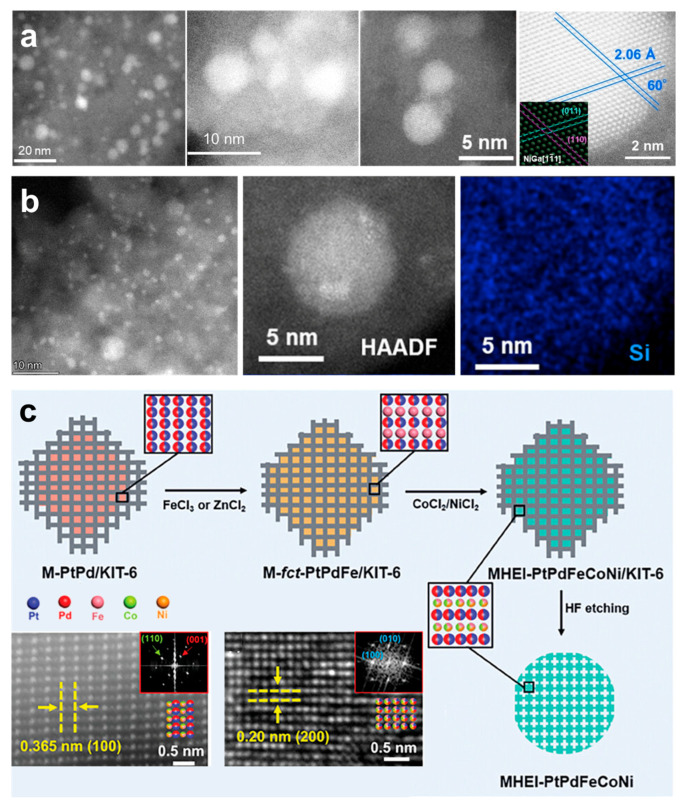
(**a**) Structure of (NiFeCu)(GaGe) HEI on SiO_2_. Reproduced with permission [22]. Copyright 2022, Wiley. (**b**) Structure of (PtCoCu)(GeGaSn) HEI on Ca-SiO_2_. Reproduced with permission [23]. Copyright 2022, American Chemical Society. (**c**) Schema illustrating the synthesis of PtPdFeCoNi HEIs. Reproduced with permission [33]. Copyright 2024, Wiley.

Ma et al. reported the synthesis of B2-NiFeCuGaGe HEI nanoparticles with CARiACT G-6, which is produced by Fuji Silysia Chemical with an amorphous structure (Figure 7a) [22]. The NiFeCuGaGe/SiO_2_ HEI nanoparticles were obtained through a pore-filling co-impregnation method, in which (NH_4_)_2_GeF_6_, Fe(NO_3_)_3_·9H_2_O, Ga(NO_3_)_3_·nH_2_O, Cu(NO_3_)_2_·3H_2_O and Ni(NO_3_)_2_·6H_2_O were used as the metal precursors. A mixed solution of the metal precursors (Ni:Ga = 1:1, Ni:Cu:Fe:Ga:Ge = 1:1:1:1) was dropwise added to CARiACTG-6. The mixture was then sealed and aged at room temperature overnight. It was then frozen in liquid nitrogen and vacuum-dried at approximately −5 °C. Following that, the sample was heated at 90 °C overnight. Finally, the NiFeCuGaGe/SiO_2_ HEI nanocatalysts were obtained by continuously treating the obtained powder at 400 °C and 700 °C in H_2_. Nakaya et al. reported the synthesis of B14-(PtCoCu)(GeGaSn) HEI nanoparticles on a Ca-modified SiO2 (Figure 7b) [23]. First, a mixed aqueous solution of Pt(NH_3_)_2_(NO_3_)_2_, Co(NO_3_)_2_·6H_2_O, Cu(NO_3_)_2_·3H_2_O, (NH_4_)_2_GeF_6_, Ga(NO_3_)_3_·nH_2_O, (NH_4_)_2_SnCl_6_, and Ca(NO_3_)_2_·4H_2_O was added to CARiACTG-6. The mixture was sealed at room temperature overnight and then frozen with liquid nitrogen. The sample was then dried at 90 °C overnight. The obtained powder was evaporated in air at 400 °C for 1 h. After that, the calcined powder was reduced under flowing H2 at 700 °C for 1 h to get (PtCoCu)(GeGaSn)/Ca-SiO_2_ HEI nanoparticles. Here, Ca may work as a structural spacer that promotes a uniform distribution of the metal components, thereby facilitating their alloying process. Wang et al. synthesized the libraries of mesoporous high-entropy intermetallics (MHEIs) on a mesoporous silica named KIT-6 (Figure 7c). The synthesis of PtPdFeCoNi/KIT-6 HEI nanoparticles involved several steps. First, PtCl_4_^2−^ and PdCl_4_^2−^ within KIT-6 are reduced by L-ascorbic acid to produce KIT-6-supported PtPd alloy nanoparticles. To obtain an ordered PtPdFe intermetallic, iron precursor was physically mixed with mesoporous PtPd/KIT-6 and reduced at 480 °C in a H_2_/N_2_ atmosphere. Finally, Co and Ni were subsequently incorporated into the alloy under the same conditions to synthesize the ordered PtPdFeCoNi MHEIs. Notably, the KIT-6 template can be etched with dilute HF [33,80,81].

#### 2.2.2. Porous CeO_2_-Confined Annealing

**Figure 8:**
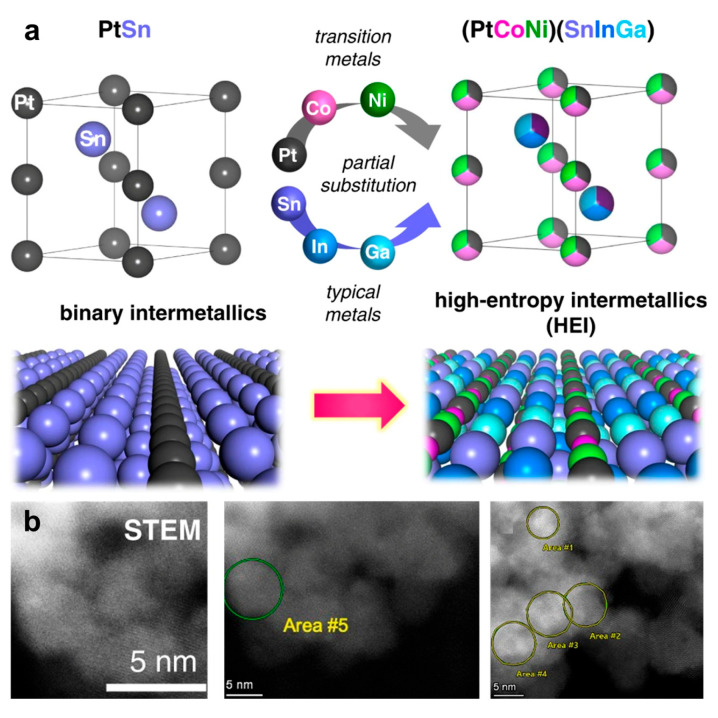
(**a**) Structure and (**b**) TEM images of (PtCoNi)(SnInGa) HEI on CeO_2_. Reproduced with permission [24]. Copyright 2022, Nature Publishing Group.

Porous CeO_2_ featuring pore channels ranging from 2 nm to 50 nm is another important material for the space-constrained annealing synthesis of HEI nanomaterials. Beside the porous channel structure, CeO_2_ exhibits unique variable valence states (Ce^3+^/Ce^4+^). The special structure will create a large number of oxygen vacancies and defect sites on its surface, which can form strong chemical bonds with metal atom and thus act as the effective metal anchoring points [82]. This enhanced metal–support interaction will effectively suppress the migration and aggregation of the HEI nanoparticles [83,84]. Additionally, this robust interaction induces electron transfer between the carrier and the HEI nanoparticles. As a result, the electronic structure of the HEI nanoparticles will be modulated, thereby optimizing their catalytic performance. Up to now, the commercial CeO_2_ named JRC-CEO-2 was used for the synthesis of HEI nanoparticles [85]. Xing et al. synthesized B8_1_-(PtCoNi)(SnInGa) HEI nanoparticles on CeO_2_ by a conventional impregnation method combined with high-temperature annealing (Figure 8a) [24]. First, a mixture, which contained the CeO_2_ support and metal precursors with an atomic ratio of 1:1:1:1:1:1, was dried at 50 °C. Then, the (PtCoNi)(SnInGa) HEI/CeO_2_ nanoparticles were prepared by calcining the dried sample in air at 500 °C and at 600 °C in H_2_ for 1 h, respectively.

#### 2.2.3. Polymer Substrate-Derived Porous Carbon-Confined Annealing

HEI nanoparticles can be also synthesized in polymer substrate-derived porous carbon-based materials. Generally, this process includes two steps. First, the metal precursors are encapsulated with a polymer substrate by mixing the metal precursors with the polymer, such as polydopamine, polyacrylonitrile (PAN), and poly(ethylene oxide)-b-poly(methyl methacrylate) (PEO b-PMMA). Second, the polymer encapsulated with metal precursors is annealed at a high temperature under reduction or an inert atmosphere to obtain porous carbon-confined HEI nanoparticles. During the annealing process, the formed rigid carbon framework acts as “nano-reactors” or “nanocages,” which will effectively confine the internal metallic species within its structure. This confinement suppresses the migration, agglomeration, and growth of the nanoparticles.

**Figure 9:**
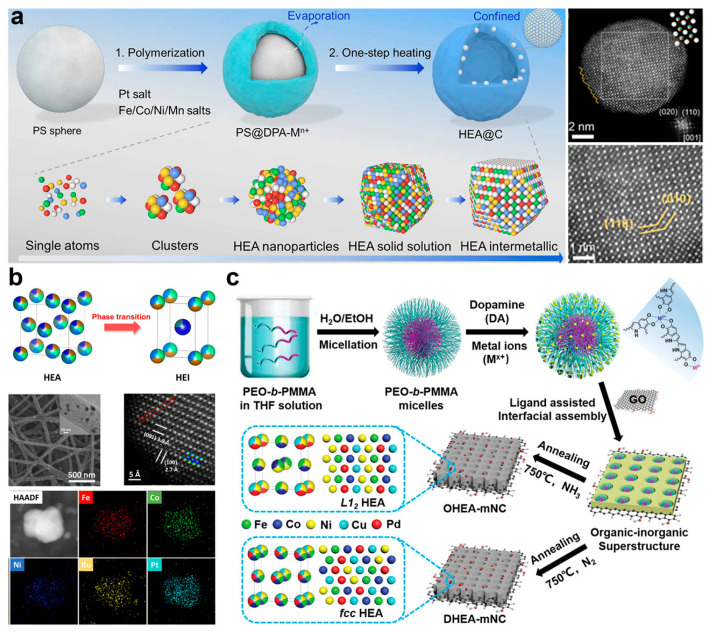
(**a**) Schematic diagram of the hollow-carbon confinement annealing. Reproduced with permission [34]. Copyright 2023, American Chemical Society. (**b**) Schematic diagram illustrating the phase transition of (FeCoNi)(RuPt) HEA to HEI. Reproduced with permission [35]. Copyright 2024, Wiley. (**c**) Schematic illustration for construction of PdFeCoNiCu HEI nanoparticles on 2D nitrogen-rich mesoporous carbon nanosheets. Reproduced with permission [36]. Copyright 2025, The Royal Society of Chemistry.

Hu et al. reported the synthesis of L1_0_-PtFeCoNiMn HEI nanoparticles with a hollow carbon substrate derived from polydopamine coated polystyrene (PS) nanospheres (Figure 9a) [34]. Initially, PS nanospheres with approximately 250 nm were synthesized through polymerization of styrene at 70 °C. These nanospheres were then mixed with metal chloride salts in a hydrochloric acid buffer solution. Following that, the PS nanospheres were coated with the polydopamine layer by introducing the polydopamine into the solution. Here, the functional groups (e.g., catechol, amines, imines) provide abundant anchoring sites to efficiently bind metal ions to the PS nanospheres (referred to as PS@DPA-Mn+). The PS@DPA-Mn+ precursor was then collected by centrifugation and annealed in an N_2_/H_2_ atmosphere. During the annealing process, the PS nanospheres were decomposed, and a carbon shell-bonded PtFeCoNiMn HEI with an average size of 5.9 nm was formed.

Hao et al. successfully synthesized the L1_0_-(PtIr)(FeMoBi) HEI nanoparticles by using PAN-derived one-dimensional carbon nanofibers (CNFs) as a confined porous space (Figure 9b) [25]. First, a uniform precursor solution was prepared by dissolving Fe(NO_3_)_3_·9H_2_O, C_10_H_14_MoO_6_, Bi(NO_3_)_3_·5H_2_O, H_2_PtCl_6_·6H_2_O, C_15_H_21_IrO_6_, and PAN in N,N-dimethylformamide. Then, PAN-based nanofibers containing (PtIr)(FeMoBi) metal salts were fabricated via electrospinning. The resulting fibers were first oxidized at 230 °C for 3 h in air, and were then graphitized at 1000 °C for 3 h in Ar. Finally, the (PtIr)(FeMoBi) HEI nanoparticles confined in CNFs were obtained. In a similar way, Liu et al. obtained the (FeCoNi)(RuPt) HEI nanoparticles confined in CNFs [35].

Zhu et al. prepared the L1_2_-PdFeCoNiCu HEI nanoparticles in nitrogen-rich mesoporous carbon nanosheets (Figure 9c) [36]. The synthesis includes two important steps. First, an organic–inorganic 2D superstructure with uniform metal distribution was synthesized by the ligand-assisted interfacial assembly of metal–catecholamine (MC)-functionalized PEO-b-PMMA composite monomicelles. Then, the PdFeCoNiCu HEI nanoparticles in nitrogen-rich mesoporous carbon nanosheets were obtained by treating the organic–inorganic 2D superstructure in NH_3_ atmosphere at 750 °C. Additionally, the amount of L1_0_ Pt-based HEI nanoparticles, including Pt(FeCoNiCu), Pt(FeCoNiZn), and Pt(FeCoNiMn), were also synthesized in a similar way [37].

Space-constrained annealing provides a useful method to synthesize HEI nanoparticles with a highly uniform size distribution, well-controlled morphology, and good dispersibility. However, the synthesis of certain confined structures such as KIT-6 is complicated and time-consuming. In addition, a key limitation is that the HEI nanoparticle characteristics are fundamentally limited by the constrained substrates. To obtain HEI nanoparticles with the desired size, a completely new substrate is required, as this method does not allow for continuously fine-tuning the particle size like the liquid-phase methods. Moreover, to obtain pure nanoparticles, the removal of the confined framework is generally required. This step may induce agglomeration and impurity of the HEI nanoparticles. Notably, the space-constrained HEI catalysts are often limited by mass transfer during the catalytic reaction, where reactants and products must diffuse through the narrow pores or cavities to access or exit the active sites. In such nano- to micro-scale confined spaces, the molecular diffusion can be significantly slowed, which will lead to low catalyst efficiency.

### 2.3. Oleylamine-Mediated Wet-Chemical Synthesis

Wet-chemical synthesis, also referred as solution-based or liquid-phase synthesis, is a class of methods in which nanomaterials are prepared in liquid solvents via chemical reactions, such as precipitation, hydrolysis, and redox reactions [86,87]. Its key characteristic lies in the chemical transformation of precursors in a solution medium, typically water or organic solvents. By carefully tuning the reaction conditions, such as temperature, surfactants, concentration, and pH, the nucleation and growth processes of the nanomaterials can be precisely controlled. This flexibility enables the synthesis of nanocrystals with a precise size, shape, composition, and structure [88,89,90,91,92,93,94]. Therefore, wet-chemical synthesis is one of the most fundamental and widely used “bottom-up” synthesis strategies in the field of nanotechnology. Thanks to its superior controllability, mild reaction conditions, and excellent scalability, it has become the preferred method for both laboratory research and industrial production of nanomaterials. Although many intermetallic compounds have been synthesized via the wet-chemical method, the preparation of HEI nanomaterials via wet-chemical methods is still in its early stages. To date, only (PdRhIrPt)Sn, (CoNiRhIrRu)Sb3, (PtRh)(BiSnSb), (NiPdPtRhIr)Zn, (NiFeCoPdPt)Zn, (NiFePdPtIr)Zn, (NiPdPtRhlr)In, (NiFeCoPdPt)In, (NiFePdPtIr)In (NiPdPtRhIr)Sn, (NiFeCoPdPt)Sn and (NiFePdPtr)Sn HEI nanoparticles have been reported, all of which have been conducted in oleylamine medium.

**Figure 10:**
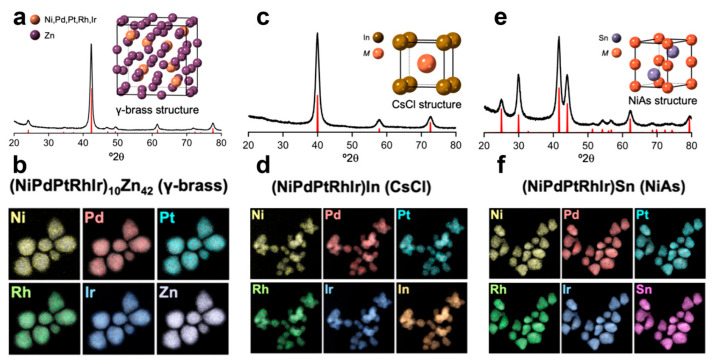
(**a**,**b**) Structure of (NiPdPtRhIr)Zn HEI nanomaterials. (**c**,**d**) Structure of (NiPdPtRhIr)In HEI nanomaterials. (**e**,**f**) Structure of (NiPdPtRhIr)Sn HEI nanomaterials. Reproduced with permission [41]. Copyright 2025, American Chemical Society.

Samuel et al. successfully synthesized a B8_2_-(PdRhIrPt)Sn HEI nanoparticle in oleylamine solution [38]. Pd(acac)_2_, Rh(acac)_2_, IrCl_4_, Pt(acac)_2_, and SnCl_2_ were first dissolved in oleylamine and sonicated to ensure complete dissolution with heating. Simultaneously, a mixture of octadecene and oleylamine was heated to 110 °C under vacuum for 1 h. The precursor solution was then slowly injected into the heated solvent mixture at 0.25 mL/min at 315 °C. Then, the solution was gradually cooled to 230 °C and rapidly quenched with a water bath. The resulting product was washed twice with a toluene–acetone mixture and centrifuged. The mechanism study revealed that the nucleation began with a cube-like Pd-rich phase with small amounts of Rh, Pt and Ir localized at the edges. As the reaction continued, Rh, Ir, Pt, and Sn were gradually incorporated, eventually co-depositing onto the growing particles. Ultimately, Sn diffused into the Pd-rich cores, resulting in a homogeneously mixed (PdRhIrPt)Sn HEI nanoparticle with a distinctive nanoflower morphology. The (CoNiRhIrRu)Sb_3_ HEI nanoparticles were synthesized in a similar condition [39]. Chen et al. successfully synthesized the B35 PtRhBiSnSb HEI nanoparticle in oleylamine [40]. First, the mixed metal precursors with a Pt+Rh/Sn+Bi+Sb ratio of approximately 1:1, cetyltrimethylammonium bromide and octadecene were dissolved in oleylamine and heated at 220 °C for 1 h with magnetic stirring. A typical “complexation–reduction–ordering” growth process was confirmed by analyzing the evolution of the crystal phase, morphology, and composition during the HEI nanoparticle formation process. Initially, a Bi-complex was formed as a template for nanoplate growth. Subsequently, the Bi complex was decomposed at elevated temperatures to generate Bi atoms. The Pt and Rh species were replaced by the Bi atoms, after which Rh and Sb atoms were substituted. Finally, (PtRh)(BiSnSb) HEI nanoplates with high uniformity were yielded through the diffusion and ordering of Pt, Rh, Bi, Sn, and Sb atoms. Soliman et al. synthesized HEI nanomaterials by a solution-based postsynthetic transformation strategy that converts HEA nanoparticles into HEIs (Figure 10) [41]. Firstly, Ni(acac)_2_, Fe(acac)_3_, Co(acac)_3_, Pd(acac)_2_, Pt(acac)_2_, Rh(acac)_3_, and IrCl_4_ were dissolved in oleylamine to prepare metal precursor solutions. The solutions were heated to 80–120 °C to ensure complete salt dissolution, which was used to inject into the heated oleylamine/octadecene solution. Depending on the target HEA composition, the solution was heated to 275 °C for NiPdPtRhIr or 315 °C for NiFeCoPdPt and NiFePdPtIr in an Ar atmosphere. For NiPdPtRhIr, 4 mL of the precursor solution was injected slowly at 0.4 mL/min for 10 min without the following reaction. For NiFeCoPdPt and NiFePdPtIr, 4 mL of precursor was injected rapidly, followed by a 1 h reaction period. The NiPdPtRhIr, NiFeCoPdPt, and NiFePdPtIr HEAs were synthesized as seeds. Next, the HEA seeds are reacted with Diphenyl zinc, InCl_3_/Li-HMDS, and SnCl_2_ in oleylamine at 320 °C for 1 h. The flask is cooled to 250 °C and then rapidly quenched in room-temperature water. The particles are washed with an acetone/toluene mixture, centrifuged, and resuspended in toluene. Then, finally synthesize (NiPdPtRhIr)_10_Zn_42_, (NiFeCoPdPt)_10_Zn_42_, (NiFePdPtIr)_10_Zn_42_ HEI nanoparticles with the γ-brass type, (NiPdPtRhlr)In, (NiFeCoPdPt)In, (NiFePdPtIr)In HEI nanoparticles with the CsCl type and (NiPdPtRhIr)Sn, (NiFeCoPdPt)Sn, (NiFePdPtr)Sn HEI nanoparticles with the NiAs type. Here, the HEA nanoparticles with homogeneous mixing were synthesized to serve as reactive seeds. The HEIs were formed by the diffusion of the post-transition metal atoms, such as Sn, In, and Zn, into the HEA seeds.

The wet-chemical method has demonstrated great potential in the synthesis of HEIs due to its controllability over reaction conditions. Nevertheless, challenges and limitations remain in the wet-chemical synthesis of HEIs. First, the synthesis mechanisms are unclear due to the complicated reaction process in solution, which is strongly affected by the precursor concentration, reaction temperature, solvent, protecting agent, etc. As a result, the current syntheses heavily rely on the empirical exploration. The general synthesis strategy of HEI nanomaterials needs to be further explored [95,96]. Furthermore, current syntheses of HEI nanomaterials are based on the media of oleylamine. However, the oleylamine molecule consists of an amino group and a long alkyl chain. The amino group contains a lone pair of electrons that can form strong coordination bonds with metal sites. In addition, the long carbon chains exhibit strong van der Waals interactions, enabling effective adsorption onto metal nanoparticles. Therefore, the surfaces of the HEI nanomaterials synthesized with oleylamine are covered by oleylamine molecules, which are hard to remove and significantly impede mass transport and charge transfer during catalytic processes. It is crucial to explore the wet-chemical synthesis routes for HEIs in non-oleylamine systems, such as aqueous or alcoholic media [97,98,99].

### 2.4. Other Methods

**Figure 11:**
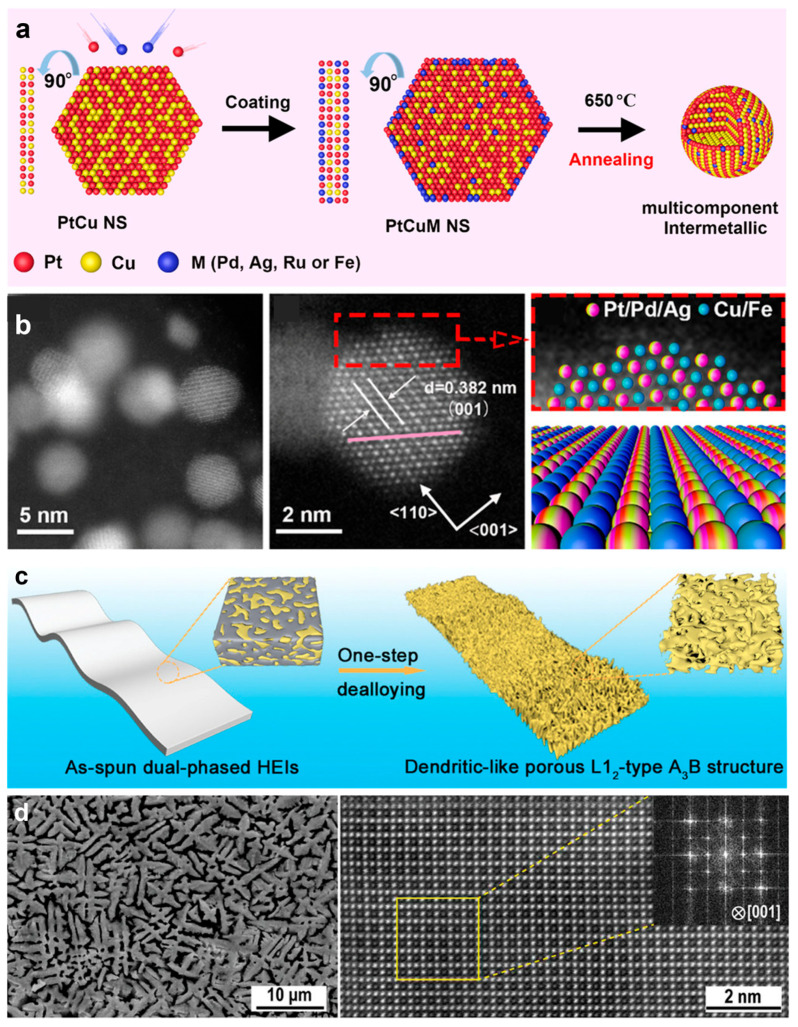
(**a**) Schematic illustration of the synthesis of PtCuPdAgRu and PtCuPdAgFe HEI nanoparticles. (**b**) Structure of PtCuPdAgFe HEI nanoparticles. Reproduced with permission from [26]. Copyright 2024, Wiley. (**c**) Schematic diagram of the dealloying process to a dendritic-like L1_2_ structure, (**d**) structure of the L1_2_-type (FeCoNi)(AlTi) HEI nanomaterials. Reproduced with permission from [42]. Copyright 2020, Wiley.

Zheng et al. report a template-based epitaxial growth method to prepare HEI nanoparticles (Figure 11a,b) [26]. To prepare the L11-PtCuPdAgFe HEI nanoparticles, ultrathin bimetallic PtCu nanosheets were firstly synthesized as the starting materials by co-reduction of Pt(acac)_2_ and Cu(acac)_2_ in a HCHO solution. Here, it is worth pointing out that the PtCu nanosheets can be converted into the L11-PtCu intermetallic structure by annealing at 500 °C. Next, the PtCu nanosheets were used as templates to epitaxially grow other metals. The PtCu nanosheets, AgNO_3_, Pd(acac)_2_, FeCl_3_, KI and ascorbic acid were mixed in the formamide solvent and heated at 130 °C for 3 h to obtain PtCuPdAgFe multicomponent nanosheets in a Teflon-lined stainless-steel autoclave. Finally, the ordered PtCuPdAgFe HEI nanoparticles were obtained by annealing the PtCuPdAgFe multicomponent nanosheets at 650 °C in a N_2_ atmosphere. Using this method, PtCuPdAgRu HEI nanoparticles were also prepared. Jia et al. synthesized dendrite-like porous L1_2_-type (FeCoNi)(AlTi) HEI nanomaterials by using a one-step chemical dealloying method (Figure 11c,d) [42]. First, a master alloy ingot with elements including Fe, Co, Ni, Al and Ti was prepared by an arc-melting method under a high-purity Ar atmosphere. Then, the alloy was re-melted under the protection of a high-purity Ar atmosphere at 1200–1500 °C. Next, the (FeCoNi)(AlTi) HEI ribbons with a dual-phased structure (Heusler and L1_2_ phases) were obtained by rapidly quenching the melt liquid on the single rotating copper wheel surface. Finally, the (FeCoNi)(AlTi) HEI ribbon with an L1_2_ phase was obtained by treating the dual-phased HEI ribbon in a 1.0 M HCl solution to remove the Heusler phase.

Overall, even though several synthesis strategies have been developed to prepare the HEI catalysts, the development of the HEI catalysts is still in the early stage. Table 2 has summarized the advantages and drawbacks of the current reported synthesis strategies. Methods such as space-constrained annealing and template-based epitaxial growth offer superior control over the crystal structure, particle size, and atomic ordering, enabling the preparation of highly uniform and well-defined materials. However, their reliance on sophisticated templates, lattice-matched substrates, and complex processing conditions significantly limits scalability and increases production costs, thereby constraining their industrial relevance. In contrast, carrier-supported annealing demonstrates better scalability and reproducibility due to simpler processing routes, yet they are often sensitive to support stability and high energy consumption. Wet-chemical synthesis methods provide excellent compositional tunability and design flexibility but suffer from issues related to synthetic complexity, ligand removal, and batch-to-batch variability. Future efforts should focus on developing more general synthesis routes that can be used to prepare plenty of HEI nanocatalysts with the desired amount to meet industrial applications.

## 3. Catalytic Applications

HEI nanomaterials have been studied in various catalytic reactions due to their unique properties, including propane dehydrogenation, alkyne semi-hydrogenation, alcohol oxidation reaction (AOR), formic acid oxidation reaction (FAOR), nitrate reduction reaction, oxygen reduction reaction (ORR) and hydrogen evolution reaction (HER)

### 3.1. Propane Dehydrogenation

Propylene is a crucial raw material in the petrochemical industry. Currently, enhancing the efficiency of propylene production from shale gas has become a hot research topic [100,101]. Selective propane dehydrogenation for propylene production is considered the most promising technology, like Fischer−Tropsch-to-olefins and methanol-to-olefins processes, due to its outstanding propylene selectivity, which meets the rising demand for propylene [102,103,104]. However, propane dehydrogenation is endothermic and requires high operational temperatures (>600 °C) to achieve adequate propylene yields, which inevitably leads to severe catalyst deactivation through coking and/or sintering within short periods [105,106]. In this scenario, design of novel propane dehydrogenation catalysts, which maintain excellent propylene selectivity and catalyst stability at high temperatures (>600 °C), would be highly beneficial for the chemical industry.

**Figure 12:**
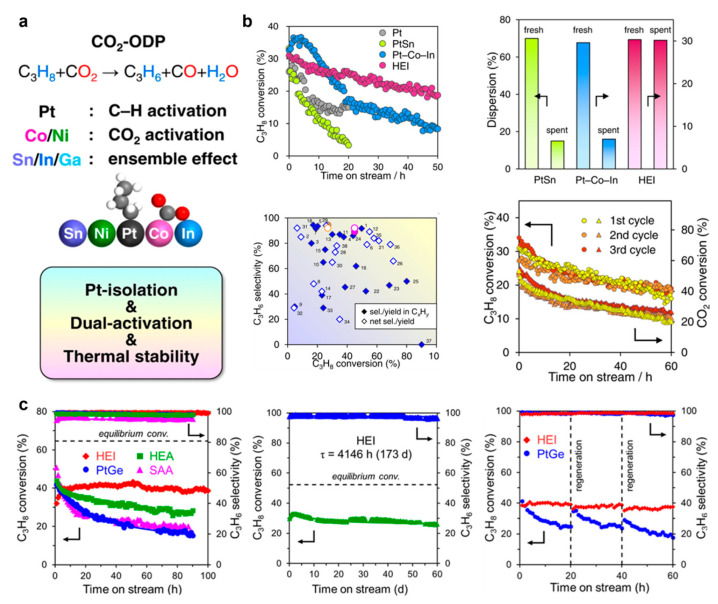
(**a**) Schematic diagram of CO_2_-propane dehydrogenation catalyzed with (PtCoNi)(SnInGa) HEI. (**b**) Catalytic performance of (PtCoNi)(SnInGa) HEI for CO_2_-propane dehydrogenation. Reproduced with permission [24]. Copyright 2022, Nature Publishing Group. (**c**) Catalytic performances of the (PtCoCu)(GeGaSn) HEI in propane dehydrogenation. Reproduced with permission [23]. Copyright 2022, American Chemical Society.

Xing et al. reported the (PtCoNi)(SnInGa) HEI nanocatalyst for propane dehydrogenation based on a PtSn intermetallic compound due to its high C_3_H_6_ selectivity [24]. Here, the Pt site was partially replaced by Ni/Co, while the Sn site in the PtSn intermetallic compound was partially substituted with In/Ga. CeO_2_ was employed as the catalytic support due to its basic nature and oxygen-releasing properties, which facilitate CO_2_ capture and combustion of the deposited carbon. The HEI/CeO_2_ catalyst exhibits 1.5- and 2-times higher specific activity and a longer catalyst life than those of the best-reported catalyst. In particular, the HEI/CeO_2_ catalyst exhibited an excellent regeneration capability, with it being able to fully recover its conversion at the reaction temperature (Figure 12a,b). DFT calculation indicates that the polymetallization of a (PtCoNi)(SnInGa) HEI can effectively isolate the Pt atoms, which effectively inhibits side effects, thus suppressing the propylene decomposition and coke formation. Meanwhile, the introduction of Ni/Co notably enhanced the CO_2_ activation, while the dopped In/Ga greatly enhanced the stability of the catalyst because of the entropic effect. Moreover, according to the result of the temperature-programmed surface reactions, the oxygen-releasing ability of CeO_2_ plays a crucial role in the coke combustion and the excellent regeneration ability. The side reactions of propane dehydrogenation, including coking, cracking, and hydrogenolysis, are sensitive to the structure of the catalysts. In particular, they easily occur when active metal–metal ensembles such as Pt−Pt sites exist. Dilution of Pt-Pt ensembles is a common strategy to design a high-performance catalyst for propane dehydrogenation.

Nakaya et al. synthesized a (PtCoCu)(GeGaSn) HEI nanocatalyst based on a PtGe intermetallic with a B14 type (Pnma) to completely isolate Pt sites, in which the Co/Cu and Ga/Sn elements were used to partially substitute the Pt and Ge atoms in the intermetallic PtGe [23]. The degree of Pt isolation can be tuned by the Pt fraction in the Pt site [Pt/(Pt + Co + Cu) ratio]. The single-atom-like Pt sites in the HEI (0.25) catalyst can effectively facilitate propylene desorption and prevent unwanted side reactions. The HEI (0.25) catalyst did not show any deactivation within 100 h and maintained 99% propylene selectivity for up to 260 h, while the PtGe catalyst exhibited rapid deactivation within 20 h due to the coke accumulation and the nanoparticle aggregation (Figure 12c). The C_3_H_6_-TPD suggested that the exceedingly weak adsorption of propylene on the HEI surface effectively suppressed the propylene decomposition and coke formation. DFT calculation results indicated that the first and second C−H scissions of propane could be selectively catalyzed by the single-atom Pt on HEIs. Furthermore, the single-atom Pt on HEIs could accelerate the propylene desorption and thus effectively inhibited the subsequent side reactions.

### 3.2. Alkyne Semi-Hydrogenation

Alkyne semi-hydrogenation is a crucial reaction for the production of polymer-grade ethylene and various fine chemicals [107,108]. Palladium-based alloy materials are the most commonly used catalysts for alkyne semi-hydrogenation. However, this reaction is often accompanied by side reactions such as over-hydrogenation and oligomerization, which result in compromised selectivity toward the target alkenes [109,110,111,112,113]. Therefore, the development of highly efficient catalysts is essential to suppress these undesirable pathways and enhance process selectivity. In recent years, HEI nanomaterials have been employed for alkyne semi-hydrogenation owing to their unique geometric and electronic properties.

**Figure 13:**
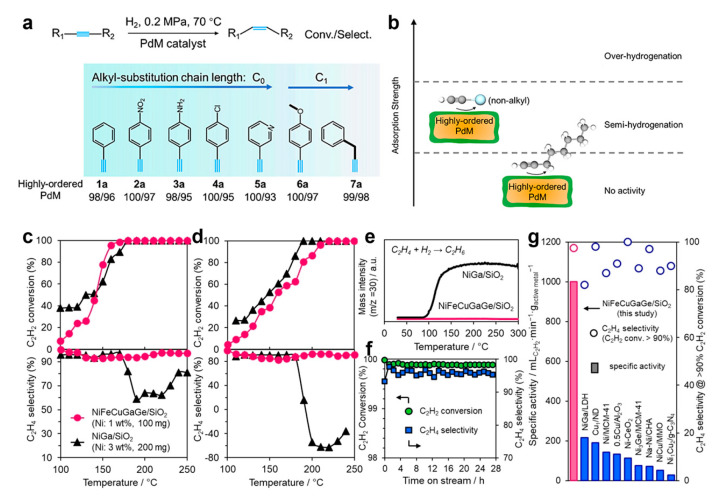
(**a**,**b**) Semi-hydrogenation of C_0_ or C_1_-alkynes over PdFeCoNiCu HEI catalysts. Reproduced with permission [36]. Copyright 2024, American Chemical Society. (**c**,**d**) Catalytic performance of NiFeCuGaGe/SiO_2_ HEl in acetylene semi-hydrogenation in the absence and presence of excess ethylene, respectively. (**e**) Temperature-programmed surface reaction results. (**f**) Stability test. (**g**) Summary of reported 3D transition metal-based selective semi-hydrogenation catalysts. Reproduced with permission [22]. Copyright 2022, Wiley.

Liu et al. reported a PdFeCoNiCu HEI nanocatalyst with an ordered core covered by a disordered shell [36]. In the semi-hydrogenation of C_0_ and C_1_ alkyl-substituted alkynes, the PdFeCoNiCu HEI catalyst demonstrated high alkene selectivity of 93–98% with virtually complete substrate conversion (Figure 13a,b). An enhanced electron density was identified at the Pd sites on the surface of PdFeCoNiCu HEI nanoparticles, as indicated by the presence of negatively charged Pd^δ−^ species confirmed through EELS and CO-DRIFTS. This electron density enhancement is ascribed to the electron redistribution stemming from the core-ordering process between surface Pd and M atoms. CV measurements confirmed that the surface adsorption of the C_0_ or C_1_-alkyl species possessed an intermediate adsorption strength due to the unique electronic structure of the HEI nanoparticles, thereby suppressing over-hydrogenation.

Ma et al. synthesized SiO_2_-supported Ni-based HEI catalysts for efficient acetylene semi-hydrogenation [22]. By incorporating multiple metals, the NiGa structure was transformed into (NiFeCu)(GaGe) HEIs, which retained the CsCl-type structure and isolated Ni atoms from the minor metal elements. The (NiFeCu)(GaGe) HEI catalyst delivered an outstanding catalytic performance. First, it achieved outstanding ethylene selectivity (98%) and acetylene conversion (99.8%). Additionally, its specific activity reached 1000 mLC_2_H_2_ min^−1^ g_Ni_^−1^ at 80% conversion, surpassing the NiGa benchmark and other 3D transition-metal catalysts by a factor of five. Notably, the catalyst maintained high ethylene selectivity for over 28 h at 190 °C, establishing unprecedented stability for a Ni-based catalyst in acetylene semi-hydrogenation (Figure 13c–g). According to the kinetic study, multimetallic alloying within the HEI framework decreased in the energy barrier of the semi-hydrogenation and improved the hydrogen supply, thus enhancing catalytic activity. Meanwhile, DFT calculations revealed that the HEI surface possesses a significantly lower energy than NiGa, which can be attributed to the surface relaxation induced by multi-metallization. This lower surface energy of the HEI phase thus leads to a weaker adsorption of ethylene, which could contribute to the observed high catalytic performance.

### 3.3. Alcohol Oxidation Reaction

Direct alcohol fuel cells have received much attention for their use of liquid alcohol fuels, which are safer and more efficient than hydrogen at room temperature and are easy to produce, store, and transport [114,115,116,117]. However, AORs, such as ethylene glycol oxidation, glycerol oxidation, ethanol oxidation, and methanol oxidation reaction, are kinetically slower processes and involve a variety of reaction intermediates that require versatility of the active site [118,119,120]. Most of the AORs are multistep reactions, in which the reaction paths are critically dependent on the surface structures of the catalysts [121]. Complete alcohol oxidation is a multielectron/proton reaction including a series of C−C bond cleavage, dehydrogenation and oxidation reactions, where the selectivity of the reaction pathway is critical to the reaction efficiency. However, the majority of catalysts, including Pd and Pt as well as their alloys, show poor CO intermediate tolerance, low selectivity of the reaction pathway, and weak C−C bond-breaking ability [122]. Therefore, it is critical to develop AOR catalysts with promoted C−C bond cleavage, high selectivity of the reaction pathway, and good CO tolerance to accelerate the applications of the direct alcohol fuel cells. HEI nanocatalysts demonstrate significant potential for AORs, owing to their tunable elemental composition, adjustable stoichiometry, and long-range ordered atomic arrangement. In particular, the atomic configuration can profoundly influence the selectivity in multi-proton/electron transfer processes [123]. This stands in contrast to solid-solution-type HEAs, which often exhibit considerable spatial heterogeneity owing to the random distribution of constituent elements with differing atomic radii. Such inherent compositional non-uniformity directly affects the distribution, abundance, and nature of catalytically active sites, resulting in diverse microenvironments and parallel reaction pathways. In comparison, ordered HEI nanocatalysts feature uniform long-range atomic ordering, which promotes a homogeneous distribution of active sites and thereby enhances reaction selectivity.

**Figure 14:**
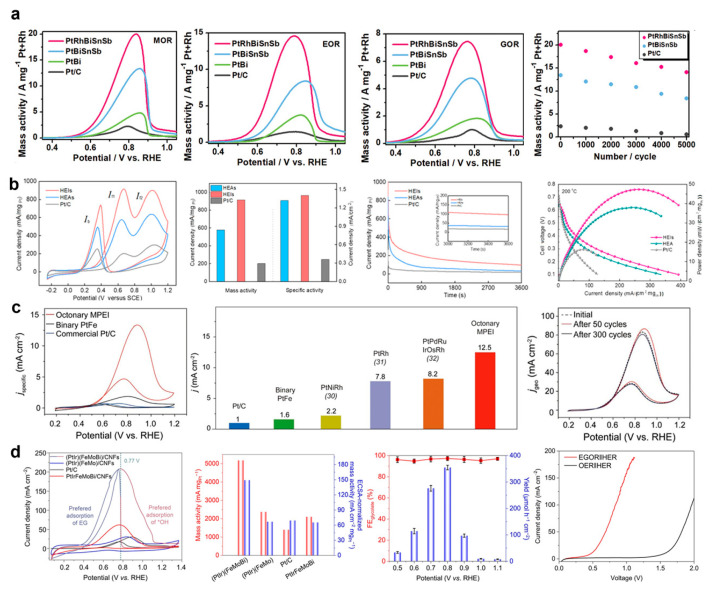
Catalyst performance of (**a**) PtRhBiSnSb HEI nanomaterials for MOR, EOR, and GOR respectively in alkaline solution. Reproduced with permission [40]. Copyright 2022, Wiley. (**b**) PtRhFeNiCu HEIs in 0.1 mol/L HClO_4_+0.2 mol/L CH_3_CH_2_OH solution. Reproduced with permission [20]. Copyright 2022, The Authors, Wiley. (**c**) (Pt_0.8_Pd_0.1_Au_0.1_)(Fe_0.6_Co_0.1_Ni_0.1_Cu_0.1_Sn_0.1_) HEI nanoparticles for EOR in 1 M KOH. Reproduced with permission [21]. Copyright 2022, AAAS. (**d**) (Ptlr)(FeMoBi) HEI in 1 M KOH with 1 M ethylene glycol. Reproduced with permission [25]. Copyright 2024, Wiley.

Chen et al. successfully synthesized a hcp-structured (PtRh)(BiSnSb) HEI nanosheet by substituting Sn and Sb atoms at the Bi sites and Rh atoms at the Pt sites of the PtBi intermetallic [40]. The PtRhBiSnSb HEI catalyst exhibited peak mass activities of 7.535, 15.558, and 19.529 A·mg^−1^ for the glycerol oxidation reaction (GOR), ethanol oxidation reaction (EOR), and methanol oxidation reaction (MOR), respectively. The catalytic performance of the (PtRh)(BiSnSb) HEI compared with the representative EOR catalysts is shown in Table 3. Moreover, the catalyst retained 70.2% of its initial activity after 5000 cycles in the MOR and demonstrated strong resistance to CO poisoning (Figure 14a). Notably, the PtRhBiSnSb HEI electrocatalyst exhibited exceptional CO poisoning tolerance during MOR, primarily attributable to its unique structural properties. CO stripping experiments revealed that CO adsorption was significantly suppressed or even completely eliminated, owing to the atomic isolation of Pt active sites and the increased Pt–Pt distances on the catalyst surface. Furthermore, the CO poisoning of Pt-based catalysts was reduced by introducing the elements with high oxygen affinity, such as Bi, Sb, and Sn. This was because the CO adsorption energy on these elements was lower, which would facilitate the oxidation of adsorbed CO to CO_2_ and promote the formation of OH adsorbates. FTIR and ATR-SEIRA spectroscopic analyses further confirmed that the PtRhBiSnSb HEI nanosheets catalyzed methanol oxidation via a CO-free pathway, exhibiting a lower onset potential than commercial Pt/C. This indicated a superior electrocatalytic performance in MOR while avoiding the accumulation of CO intermediates and associated catalyst poisoning. DFT calculations revealed that the incorporation of Rh modulated the d-band center position of the PtRhBiSnSb HEI, and accelerated electron transfer, thereby enhancing the overall electrocatalytic activity. The optimized electronic structure further improved the durability and selectivity of the catalysts along the CO_2_ pathway in AORs.

Wang et al. prepared a PtRhFeNiCu HEI catalyst through the phase transformation of a disordered solid solution at high temperature [20]. During the transformation process, compressive strain was introduced into the alloy structure. The PtRhFeNiCu HEI exhibited outstanding electrocatalytic activity and stability in the EOR. The highest mass activity of the PtRhFeNiCu HEI catalyst was 914 mA·mg^−1^, which was significantly higher than that of its solid solution type (578 mA·mg^−1^) and commercial Pt/C catalysts (202 mA·mg^−1^). In a high-temperature polybenzimidazole-based direct ethanol fuel cell, the HEI catalyst delivered a peak power density of 47.50 mW·cm^−2^, which was 2.97 times that of the Pt/C (Figure 14b). Both online gas chromatography and DFT calculations confirmed that the HEI exhibited an improved C–C bond breaking capability and superior CO tolerance performance. The adsorption configuration of intermediate CO is a critical factor governing the kinetics of EOR. The atomic ordering degree of the alloy nanocatalysts strongly affects the CO adsorption behavior during the reaction. For HEI, Rh atoms are isolated by surrounding Pt atoms. CO preferentially binds to the top site of adjacent Pt atoms, which is also the energetically most favorable adsorption site for the CH_2_*CO* intermediate. In contrast, the random atomic distribution in HEAs permits the formation of adjacent Rh–Rh pairs, and the strong adsorption capability of neighboring Rh atoms induces CO adsorption on the Rh–Pt bridge site, deviating from the most stable Rh top site configuration in CH_2_*CO*. This difference in adsorption configuration leads to a relatively lower transition state energy on HEIs compared with that on HEAs. Therefore, the ordered structure of HEIs effectively lowers the activation energy barrier by altering the adsorption geometry of key reaction intermediates, and improves the overall ethanol oxidation performance.

Cui et al. successfully prepared L1_0_-structured (PtPdAu)(FeCoNiCuSn) HEI nanoparticles with sizes of around 4 to 5 nm incorporating up to eight distinct metallic elements [21]. Structural characterization confirmed a well-defined dual-sublattice configuration, in which noble and non-noble metals occupied distinct crystallographic sites. Benefiting from their multi-component nature, ordered structure, and nanoscale dimensions, the (PtPdAu)(FeCoNiCuSn) HEI nanocatalyst exhibited exceptional electrocatalytic activity and stability in the EOR. The peak mass activity of (PtPdAu)(FeCoNiCuSn) HEI nanoparticles was eight times higher than the PtFe intermetallic compound. Meanwhile, the HEI catalyst demonstrated outstanding durability, which was attributed to the unique multi-elemental ordered intermetallic structure with an ultrasmall particle size (Figure 14c).

Hao et al. successfully synthesized L1_0_-(PtIr)(FeMoBi) HEI nanoparticles that had a long-range order and atomic-scale short-range disorder by introducing Bi and Mo into the B site of the FePt parent metal [25]. The (PtIr)(FeMoBi) HEI catalyst showed a high mass activity of 5.2 A mg_pt_^−1^ and a 95% Faradaic efficiency toward glycolic acid in the ethylene glycol oxidation reaction, surpassing most reported catalysts. Moreover, when the catalyst is integrated into an ethylene glycol oxidation reaction/HER coupled system, the cell voltage only requires 0.48 V to reach a current density of 10 mA cm^−2^, which is 1080 mV lower than that for a traditional oxygen evolution reaction/HER system (1.56 V), suggesting higher electrocatalytic efficiency and lower energy consumption in the cell. The catalytic activity of a (PtIr)(FeMoBi) HEI nanocatalyst was maintained during the 30 h stability test, while the Pt/C catalyst drastically decreased after 3 h (Figure 14d). DFT calculations and in situ experimental studies reveal that the ordered arrangement of Pt/Ir and Fe/Mo/Bi pairs within the HEI structure facilitates the synergistic adsorption of ethylene glycol and OH species, thereby lowering the energy barriers for both dehydrogenation and OH recombination steps. Moreover, the disruption of contiguous Pt sites suppressed the C–C bond cleavage through the C_1_ pathway and therefore promoted the highly selective production of glycolic acid.

### 3.4. Formic Acid Oxidation Reaction

FAOR, which is the key anodic reaction in direct formic acid fuel cells, is designed to electro-oxidize formic acid molecules efficiently and completely into carbon dioxide, thereby releasing protons and electrons [130,131,132,133]. This reaction is commonly described by a dual-pathway mechanism [134]. The first pathway is the dehydrogenation route, in which formic acid undergoes direct dehydrogenation to produce CO_2_. This route exhibits high reactivity and avoids the formation of poisoning intermediates. The second pathway is the dehydration route, in which the formic acid is first dehydrated to form adsorbed CO and then subsequently oxidized. The CO generated in the latter pathway can strongly adsorb onto active sites, particularly on platinum-based catalysts, leading to significant catalyst poisoning and rapid performance degradation [135,136,137,138]. Therefore, the primary objective in high-performance electrocatalyst design for FAOR is the selective promotion of the direct pathway over the indirect route, which is essential for lowering the reaction overpotential and boosting both activity and stability.

**Figure 15:**
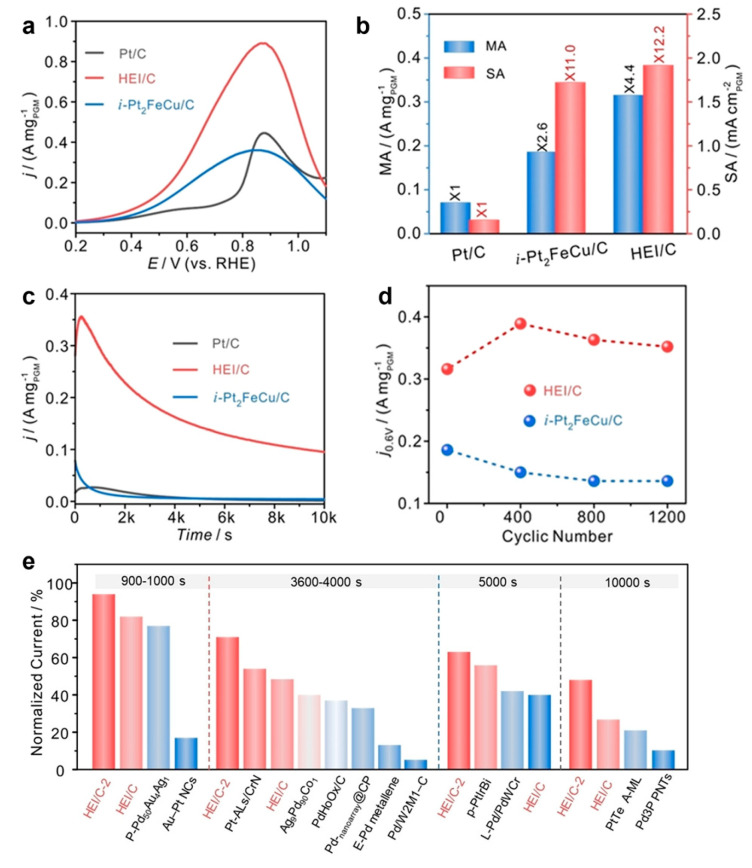
(**a**) LSV curves of (PtPdIrRu)_2_FeCu HEI/C electrocatalysts in 0.5 M H2SO4 + 0.5 M HCOOH. (**b**) Mass activity and specific activity for FAOR at 0.6 V. (**c**) Chronoamperometry test in 0.5 M H_2_SO_4_ + 0.5 M HCOOH. (**d**) Mass activity of (PtPdIrRu)_2_FeCu HEI/C at 0.6 V after potential cycling for different cycles. (**e**) Summary of the retention rates of current during the chronoamperometry tests on different reported catalysts. Reproduced with permission [18]. Copyright 2024, Wiley.

Shen et al. successfully synthesized L1_0_-ordered (PtPdIrRu)_2_ FeCu HEI nanoparticles with an average size below 2 nm [18]. These nanoparticles contained diluted Pt/Pd/Ir/Ru active sites and partially negatively charged Pt species. The HEI catalyst demonstrated significantly enhanced performance for the FAOR, achieving mass and specific activities that were 4.4 times and approximately 10 times higher compared to the pure Pt. Moreover, the HEI catalyst exhibited a 1.7-fold-higher mass activity compared to the Pt_2_FeCu intermetallic compound. Remarkably, the HEI catalyst showed markedly slower current decay even after 10,000 s of the chronoamperometry test. It also retained an approximately 11.4% increase in mass activity after 1200 cycles. In contrast, the intermetallic Pt_2_FeCu showed a dramatic decrease in mass activity, with about 26.9% of activity lost after 1200 cycles (Figure 15a–e). In situ infrared spectroscopy and DFT calculations further demonstrated that CO adsorption on the Pt site of the HEI was significantly weakened, while the energy barrier for CO diffusion across different sites was much higher than that on pure Pt surfaces, thus effectively suppressing CO accumulation and catalyst poisoning.

### 3.5. Nitrate Reduction Reaction

Electrocatalytic reduction of nitrate to ammonia has recently garnered significant research interest as it offers a promising pathway for simultaneous remediation of nitrate-contaminated water and synthesis of value-added chemicals. This conversion involves multiple proton-coupled electron transfer steps at the catalyst surface, starting with the adsorption and activation of NO^3−^ and H_2_O molecules, followed by the transformation of multiple intermediates along with protons (*H), ultimately leading to the production of NH3. However, the reaction suffers from complex pathways, slow kinetics, and strong competition from the hydrogen evolution reaction, which often lead to unsatisfactory Faradaic efficiency and product selectivity [139,140]. Therefore, the development of highly efficient and selective electrocatalysts represents a central challenge for enabling practical applications.

**Figure 16:**
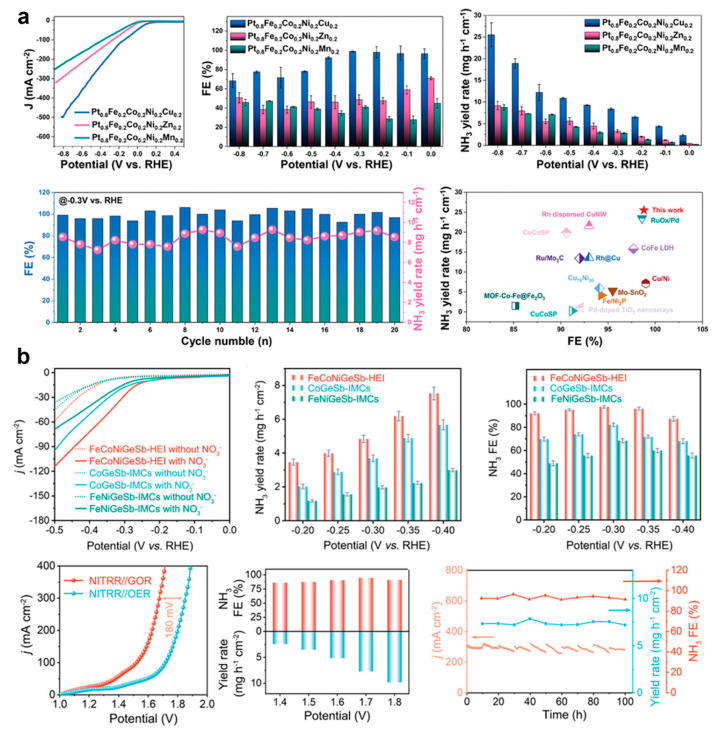
Electrocatalytic NO^3^−RR performances of (**a**) Pt_0.8_Fe_0.2_Co_0.2_Ni_0.2_Cu_0.2_ HEI. Reproduced with permission [37]. Copyright 2024, Wiley. (**b**) FeCoNiGeSb HEI. Reproduced with permission [31]. Copyright 2025, Wiley.

Zhu et. al. reported L1_0_-ordered Pt-based HEI nanoparticles on a two-dimensional nitrogen-doped mesoporous carbon, where Pt atoms occupied the A sites and transition metals (Fe, Co, Ni, Cu et.al.) occupied the B sites [37]. The Pt-based HEI catalysts demonstrated a remarkable catalytic performance and structural stability under both acidic and basic conditions, outperforming most of the reported NRA catalysts. In particular, the Pt_0.8_Fe_0.2_Co_0.2_Ni_0.2_Cu_0.2_ HEI catalyst exhibited a high Faradaic efficiency (>97%) and an ammonia production rate of 25.52 mg h^−1^ cm^−2^ in 1 M KOH, which was 2.78 and 2.92 times higher than that of Pt_0.8_Fe_0.2_Co_0.2_Ni_0.2_Zn_0.2_ and Pt_0.8_Fe_0.2_Co_0.2_Ni_0.2_Mn_0.2_, respectively (Figure 16a). The atomic structural analysis, in situ characterizations and DFT calculation demonstrated that the outstanding performance originated from the multi-site synergistic nature of the L1_0_ HEI catalyst. During the reaction, the NOx species were effectively adsorbed and activated on the Pt–X bridge sites of the HEI catalysts, while the H* were mainly adsorbed on the isolated Pt sites.

Ma et al. reported an ordered FeCoNiGeSb HEI nanoparticle with an hcp structure, where the Fe, Co, and Ni atoms occupied the Ni-column sites, while the Ge and Sb atoms resided at the Sb-column sites [31]. The FeCoNiGeSb HEI catalyst showed a high NH_3_ generation rate of 7.5 mg h^−1^ cm^−2^ at –0.40 V and a 97.6% Faradaic efficiency at –0.30 V in 1.0 M KOH. The degradation of the high NH_3_ production rate and Faradaic efficiency were negligible when compared with the initial state after a 12 h test (Figure 16b). Furthermore, when applied in membrane electrode assemblies coupled with the glycerol oxidation reaction, the NH_3_ yield was maintained at around 9.8 mg h^−1^ cm^−2^ at 1.8 V during 100 h of operation. DFT calculations and experimental characterization revealed that the Co sites were the key active centers for NRA, which effectively reduced the energy barrier of the rate determining step (*NO → *NOH). In addition, the Fe and Ni atoms synergistically modulated the electronic structure of the Co sites, which enhanced the adsorption of nitrate intermediates. Meanwhile, the incorporation of Ge and Sb effectively suppressed the competing HER.

### 3.6. Oxygen Reduction Reaction

ORR represents a cornerstone process in electrochemical energy conversion technologies, most notably in fuel cells and metal–air batteries [141,142]. The ORR is a complex multi-step reaction. There are two principal pathways for ORR. One is the four-electron pathway, which is the most desirable path for fuel cells, as it reduces oxygen directly to H2O with high efficiency. Another is the two-electron pathway, which is less efficient and produces a H_2_O_2_ byproduct, which can damage the catalyst and other cell materials. Based on the mechanisms of ORR, the surface property of an electrocatalyst strongly affects the ORR pathways and the final products. In addition, the ORR suffers from intrinsically slow kinetics due to the high energy barrier associated with the O=O bond cleavage, which needs a high overpotential. The slow ORR kinetics hinder the application of the fuel cell. Currently, Pt-based catalysts remain the best catalysts for ORR, demonstrating excellent activity and durability during the reaction [143,144]. Since platinum resources are scarce and costly, developing efficient catalysts with a lower platinum content or replacing platinum with abundant and cheap metals is an important research topic.

**Figure 17:**
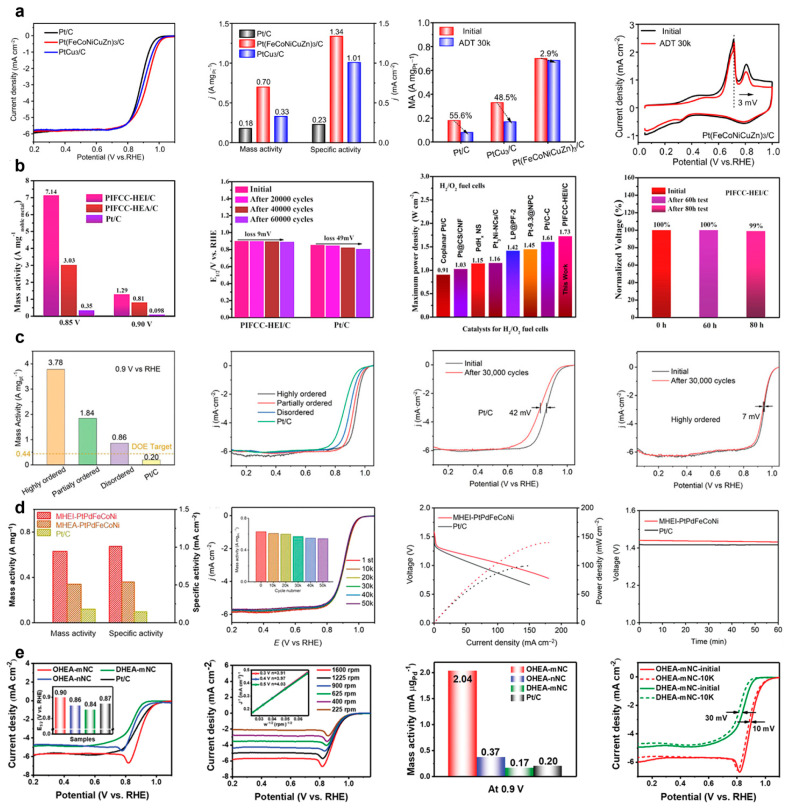
ORR catalytic performance of (**a**) Pt(FeCoNiCuZn)_3_ HEI nanoparticles in 0.1 M HClO_4_. Reproduced with permission from [27]. Copyright 2023, Elsevier. (**b**) PtIrFeCoCu HEI nanoparticles in 0.1 M HClO_4_ and H_2_/O_2_ fuel cells. Reproduced with permission [30]. Copyright 2023, American Chemical Society. (**c**) Pt_4_FeCoCuNi HEI nanoparticles in 0.1 M HClO_4_. Reproduced with permission [32]. Copyright 2023, Wiley. (**d**) PtPdFeCoNi HEI in 0.1 M KOH and the Zn-air battery performance. Reproduced with permission [33]. Copyright 2024, Wiley. (**e**) PdFeCoNiCu HEI nanoparticle in 0.1 M KOH. Reproduced with permission [36]. Copyright 2022, Wiley.

Zhang et al. constructed Pt(FeCoNiCuZn)_3_ HEI nanoparticles with a well-defined L1_2_-ordered structure [27]. Pt(FeCoNiCuZn)_3_/C delivers a remarkable ORR mass activity of 0.70 A mg_pt_^−1^ in 0.1 M HClO_4_, which is 2.1 and 3.9 times greater than PtCu_3_/C and Pt/C, respectively. After 30,000 cycles in the range of 0.6–1.0 V, the half-wave potential shifted only marginally by 2 mV, with a mass activity retention of 97.1%, significantly outperforming the severe degradation observed for Pt/C and PtCu_3_/C. Even after 10,000 cycles in the high potential between 1.0 and 1.5 V, approximately 70% of the initial mass activity was retained. Moreover, when employed as a cathode in high-temperature proton exchange membrane fuel cells (PEMFCs) operated at 160 °C, the Pt(FeCoNiCuZn)_3_ HEI nanocatalyst maintained the stable performance over 150 h of continuous operation (Figure 17a). The catalytic performance of the Pt(FeCoNiCuZn)_3_ HEI compared with representative ORR catalysts is shown in Table 4. Structural characterization and XPS analysis revealed that the exceptional stability originated from the enhanced phase stability of the high-entropy intermetallic structure, which effectively suppressed the dissolution of non-noble metals and particle aggregation. Concurrently, the downshift of the Pt d-band center optimized the adsorption energy of oxygen intermediates, thereby improving the intrinsic ORR activity.

Feng et al. obtained L10 ordered PtIrFeCoCu HEI nanoparticles (~6 nm) [30]. The PtIrFeCoCu HEI/C catalyst demonstrated outstanding ORR activity in 0.1 M HClO_4_. The current density of 7.14 A mg^−1^ was achieved at 0.85 V, which was 20.4 times higher than the commercial Pt/C. The half-wave potential only shifted 9 mV after 60,000 cycles, indicating the exceptional structural and compositional stability. The PEMFC fabricated with the PtIrFeCoCu HEI nanocatalyst showed a high peak power density of 1.73 W cm^−2^ under 1.0 bar back pressure. Moreover, the PEMFC exhibited only 1% voltage decay after 80 h of operation at 1 A cm^−2^, which was among the highest reported performances (Figure 17b). DFT calculations and experimental results demonstrated that the exceptional ORR performance and fuel cell activity of the PtIrFeCoCu HEI nanoparticles originated from their highly active facets. Notably, the (001) facet exhibited the best performance for the ORR, which could be attributed to it having the lowest activation barriers, an optimal d-band center position, and a favorable electronic structure.

Wang et al. synthesized Pt_4_FeCoCuNi nanocrystals with different degrees of ordering on a sulfur-doped carbon support [32]. The highly ordered Pt_4_FeCoCuNi catalyst achieved an ORR mass activity of 3.78 A mgPt^−1^ at 0.90 V in acidic solution, which was 2.1, 4.4, and 18.9 times higher compared to the partially ordered catalysts, disordered catalysts, and commercial Pt/C, respectively. Its half-wave potential (0.943 V) also significantly surpassed those of the control catalysts. Moreover, the half-wave potential of the highly ordered Pt_4_FeCoCuNi HEI catalyst exhibited only a 7 mV negative shift after 30,000 cycles in an accelerated durability test (Figure 17c). The measured valence band spectra demonstrated that the d-band center of Pt was upshifted with increasing structural order, which led to an optimization in the adsorption strength of oxygen species and a mitigation of excessively strong oxygen binding, thereby enhancing the ORR activity.

Wang et al. reported the synthesis of an L1_0_-ordered PtPdFeCoNi HEI catalyst [33]. The highly ordered PtPdFeCoNi catalyst demonstrated a remarkable mass and specific activities of 0.63 A mg^−1^ and 1.01 mA cm^−2^ in the alkaline ORR, respectively, which are 1.85/5.25 and 1.87/7.21 times greater than the disordered PtPdFeCoNi HEA and the commercial Pt/C. Moreover, 87.3% of the catalytic activity was maintained after 50,000 cycles, indicating the superior stability of the PtPdFeCoNi HEI catalyst. The peak power densities of the rechargeable zinc–air battery assembled with the PtPdFeCoNi HEI catalyst and with the commercial Pt/C were 139.8 mW cm^−2^ and 99.8 mW cm^−2^, demonstrating the superior performance of the PtPdFeCoNi HEI catalyst (Figure 17d). DFT calculations revealed that the combination of the multi-components and the ordered intermetallic structure could effectively regulate the surface electronic structure of a catalyst and thus enhanced the ORR catalytic activity and stability.

Zhu et al. reported an ordered L12 PdFeCoNiCu HEI nanoparticle catalyst on a 2D N-rich mesoporous carbon nanosheet, in which Fe/Co and Pd/Cu occupied the face-center and the vertex sites, while Ni was randomly distributed [36]. The PdFeCoNiCu HEI catalyst demonstrated a half-wave potential of 0.90 V, a Tafel slope of 55 mV dec^−1^, and the largest mass activity of 2.037 mA μgPd^−1^ for the alkaline ORR. Moreover, the half-wave potential was only shifted by 10 mV after 10,000 cycles (Figure 17e). The electrochemical test results combined with the structural characterizations revealed that the mass transport and electron transfer were accelerated by the 2D mesostructure support. Furthermore, DFT calculation indicated that the L1_2_-ordered structure optimized the adsorption behaviors of the reaction intermediates, which further enhanced the ORR performance.

### 3.7. Hydrogen Evolution Reaction

HER is a fundamental cathodic process in water electrolysis, aimed at efficiently and economically reducing protons or water molecules to hydrogen gas under an applied potential. As a classic model in fundamental electrochemistry, HER serves as a critical bridge connecting renewable energy sources with green hydrogen production [151,152]. The overall efficiency and economic viability of HER are highly dependent on the electrocatalyst employed. Catalysts based on platinum group metals and their derivatives are the most effective HER catalysts, owing to their low activation barriers for hydrogen desorption [153,154]. Nevertheless, the scarcity and high cost of Pt and other platinum group metals limit their large-scale use for sustainable hydrogen production. Consequently, reducing the loading of noble metals or ideally replacing them entirely with earth-abundant non-noble alternatives that exhibit high activity and durability is a critical step toward realizing a sustainable hydrogen economy [155].

**Figure 18:**
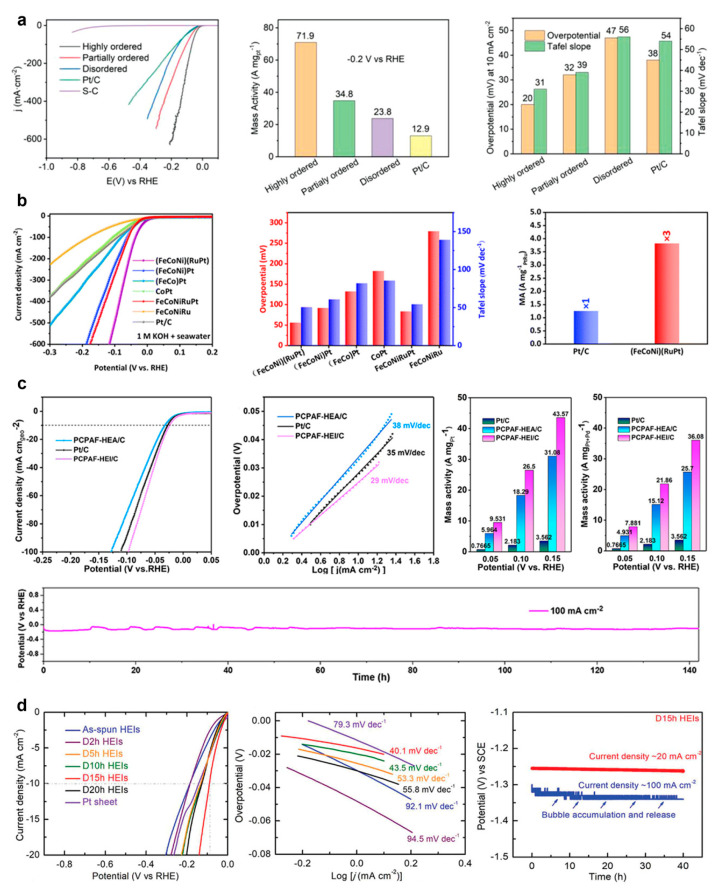
HER catalytic performance of (**a**) Pt_4_FeCoCuNi HEI in 1 M KOH solution. Reproduced with permission [32]. Copyright 2023, Wiley. (**b**) (FeCoNi)(RuPt) HEI in 1.0 M KOH + seawater. Reproduced with permission [26]. Copyright 2024, Wiley. (**c**) PtCuPdAgFe HEI in 0.5 M H_2_SO_4_ solution. (**d**) FeCoNiAlTi HEI in 1.0 M KOH solution. Reproduced with permission from [42]. Copyright 2020, Wiley.

For the alkaline HER, the highly ordered Pt_4_FeCoCuNi HEI catalyst showed a mass activity of 71.9 A mgPt^−1^ and a current density of 10 mA cm^−2^ at an overpotential of 20 mV, which were 2.1, 3.0, and 5.6 times greater than those of the partially ordered sample, disordered sample, and commercial Pt/C, respectively (Figure 18a) [32]. The catalytic performance of Pt_4_FeCoCuNi HEI compared with representative HER catalysts is shown in Table 5. In an alkaline seawater electrolyte, the (RuPt)(FeCoNi) HEI catalyst exhibited a low overpotential of 56 mV to reach 200 mA cm^−2^, a Tafel slope of 50.4 mV dec^−1^, and a mass activity of 3.8 A mg_pt+ru_^−1^ [35]. Moreover, the (RuPt)(FeCoNi) HEI catalyst was operated reliably for over 700 h at 1 A cm^−2^ (Figure 18b). In situ electrochemical Raman spectroscopy proved that the HEI contained multi-metallic active sites that facilitated water dissociation, thereby promoting the HER process. Meanwhile, the ordered intermetallic structure with the protective carbon coating ensured that the catalyst had superior corrosion resistance in seawater and high structural stability.

Zheng et al. successfully synthesized L1_0_ PtCuPdAgFe HEI nanoparticles for HER in H_2_SO_4_ solution [26]. Remarkably, the PtCuPdAgFe HEI nanocatalyst achieved an overpotential as low as 24 mV at 10 mA cm^−2^, while its mass activity of 7.881 A mg^−1^ was over 10 times greater than the commercial Pt/C. Moreover, the PtCuPdAgFe HEI catalyst maintained the performance for over 140 h at 100 mA cm^−2^. DFT calculations revealed that the ΔGH* on Pt sites of the PtCuPdAgFe HEI catalyst was closer to zero than that on pure Pt, which indicated an optimized hydrogen adsorption on the PtCuPdAgFe HEI catalyst (Figure 18c). XPS and XAFS analyses confirmed the downshift of the d-band center for the PtCuPdAgFe HEI catalyst compared to the pure Pt, leading to weakened hydrogen adsorption on Pt sites and facilitating the HER kinetics. Jia et al. reported a dendrite-like porous FeCoNiAlTi HEI nanocatalyst featuring an L1_2_-ordered intermetallic structure, which exhibited a superior catalytic performance for HER [42]. To reach 10 mA cm^−2^, the overpotential for the FeCoNiAlTi HEI nanocatalyst was 88.2 mV under alkaline conditions. The Tafel slope was 40.1 mV dec^−1^, rivaling those of noble-metal-based benchmarks (Figure 18d). Density functional theory calculations demonstrated that the intrinsic site-isolation effect offered by the L1_2_-type ordering structure stabilized the H_2_O/H* adsorption–desorption equilibria, thus substantially decreasing the reaction energy barrier and enhancing hydrogen evolution kinetics.

## 4. Conclusions and Outlook

In this review, the recent emerging synthesis strategies for HEI nanomaterials, such as carrier-supported annealing, space-confined annealing, oleylamine-mediated wet-chemical methods, etc., were summarized. In particular, the function of support and the preparation mechanism were classified and highlighted. Through these discussions, we hope to provide new insights and perspectives that may inspire the future development of HEI nanomaterial synthesis. Furthermore, the catalytic applications of the newly discovered HEI nanomaterials were also summarized, such as propane dehydrogenation, alkyne semi-hydrogenation, ORR, HER, and AOR. Impressively, benefiting from their unique structural and compositional characteristics, HEI nanomaterials have exhibited considerable promise in several critical catalytic reactions, particularly in key electrochemical reactions. Therefore, the rational design of high-performance HEI nanomaterials has come to be recognized as a prominent research frontier in materials science and catalysis.

Despite remarkable progress, current research on HEI nanomaterials still lacks a comprehensive fundamental understanding of the synthesis and catalytic mechanisms. Efforts to realize the mass production of HEI nanomaterials and their broad practical application still face numerous challenges. Here, we propose several potential research aspects for the preparation and catalytic applications of HEI nanomaterials based on current research progress.

The precise and diversified synthesis strategies still need to be explored. While initial progress has been made in synthesizing HEIs, precise control over their composition, size, morphology, and crystal structure remains challenging. Since the support materials critically determine the formation pathways of HEIs, designing innovative functional supports, such as porous carbons, MXenes, and conductive metal oxides, becomes essential. Moreover, elucidating how their surface chemistry and defect engineering regulate HEI nucleation, growth, and ordering will be key to advancing rational synthesis. Surface ligand engineering, which provide the functional groups on support surfaces and can selectively adsorb specific metal precursors, is a promising way to precise control the HEI size, morphology, and exposed facets. In addition, developing mild, energy-efficient, and controllable synthesis routes is essential to overcome conventional thermodynamic and kinetic limitations. For instance, advanced liquid-phase methods may allow the controlled preparation of HEIs at or near room temperature. Finally, most of the current synthesis methods are limited to the laboratory scale, with a low yield and high cost, hindering commercial applications. Future efforts should prioritize scalable, energy-saving, and reproducible mass production techniques.

The composition of the HEI catalysts needs to be extended. Current research on HEIs has largely focused on a limited set of transition metals, such as Fe, Co, Ni, Pt, and Pd. Future studies should actively incorporate rare-earth and alkaline-earth metals, which can create unique active sites with distinct electronic structures and coordination environments. Moreover, the vast compositional space of HEIs renders traditional trial-and-error approaches ineffective. AI tools, such as deep learning, high-throughput computation, and data mining, can analyze existing experimental and theoretical data to simulate the electronic structures of HEIs and predict their catalytic behaviors. This data-driven strategy will help establish complex structure–property relationships linking the composition, structure, and performance, ultimately enabling the inverse design of optimal HEI catalysts tailored for specific reactions.

The catalytic mechanisms need to be systematically investigated in the multi-scale level. The complex interplay among multiple elements in HEIs makes it extremely challenging to delineate how structure governs catalytic performance. Therefore, in situ characterization techniques are essential. For instance, in situ X-ray absorption spectroscopy can track the evolution of the electronic structure and local coordination of each element during reactions. In situ transmission electron microscopy enables the direct observation of dynamic changes in surface structure and morphology, while aberration-corrected electron microscopy and electron tomography offer an atomic-scale resolution of the three-dimensional structure of active sites. In addition, integrating theoretical simulations, such as density functional theory (DFT), with the above experimental approaches allows the construction of realistic structural models. Calculating reaction pathways, intermediate adsorption energies, and the electronic density of states holds promise to uncover the mechanisms of multi-element synergistic catalysis. This will help clarify why specific elemental combinations and ordered crystal lattices lead to an exceptional catalytic performance, ultimately guiding the rational design of next-generation HEI catalysts.

More catalytic applications need to be explored. The current catalytic applications of HEIs are predominantly concentrated in electrocatalysis, including reactions such as ORR, HER, and AOR. Future research should shift the focus from simple gas evolution reactions to more complex electrocatalytic processes that involve multi-electron transfer and C–C coupling. Furthermore, the application of the HEIs can be extended to thermal catalysis under harsh conditions, including high temperature, high pressure, and corrosive atmospheres. While numerous high-performing HEI catalysts have been reported at the laboratory scale, their commercial adoption remains challenging. Future research needs pay more attention to the long-term operation stability, the poisoning resistance, and the mechanisms of performance decay under real operating conditions.

In summary, HEI nanomaterials have opened up new avenues for designing high-performance catalytic materials. With continuous advances in synthetic methodology, characterization techniques, and theoretical modeling, it is expected that this emerging class of materials will play a crucial role in challenging catalytic applications.

## Figures and Tables

**Table 1:** Summary of the reported HEIs.

Method	HEI Formula	Carrier	Crystal Structure	Reference
Carrier-Supported Annealing	(PtPdIrRu)_2_(FeCu)	Carbon black (Vulcan XC-72)	B2	[18]
	Pt(FeCoNiCuZn)_3_	Acid-treated carbon black (Vulcan XC-72)	L1_2_	[27]
	Pd(FeCoNiCu)	Carbon black (VulcanXC-72R)	B2	[28]
	(PtRhRuPdIr)In	Carbon black (VulcanXC-72R)	B2	[29]
	(PtIr)(FeCoCu)	Carbon black (Ketjen-Black)	L1_0_	[30]
	(NiFeCo)(GeSb)	Carbon black (Ketjen-Black EC-300J)	B8_1_	[31]
	Pt_4_(FeCoNiCu), Pt_4_(FeCoNiMn), Pt_4_(FeCoCuMn), Pt_4_(FeNiCuMn), Pt_4_(CoNiCuMn), Pt_5_(FeCoNiCuMn)	Sulfur-doped carbon	L1_0_	[19,32]
	(PtRh)(FeNiCu)	Multiwalled carbon nanotubes	B2	[20]
	Pt(FeCoNiCu), (PtPdAu)(FeCoNiCuSn)	Carbonized wood	L1_0_	[21]
	(PtPdAu)(FeCo)	Carbonized wood	L1_2_	[21]
Space-Constrained Annealing	(NiFeCu)(GaGe)	SiO_2_	B2	[22]
	(PtCoCu)(GeGaSn)	Ca−SiO_2_	B14	[23]
	(PtPd)(FeCoNi), (PtPd)(FeCoZn), (PtPd)(FeCoGa), (PtPd)(ZnCoNi), (PtPd)(ZnCoGa), (PtRh)(FeCoNi), (PtPdRh)(FeCoNi), (PtPdRh)(FeCoNiGa), (PtPdRh)(FeCoNiGaMn),	Mesoporous silica (KIT-6)	L1_0_	[33]
	(PtCoNi)(SnInGa)	CeO_2_	B8_1_	[24]
	Pt(FeCoNiMn)	Hollow carbon	L1_0_	[34]
	(PtIr)(FeMoBi)	1D carbon nanofiber	L1_0_	[25]
	(PtRu)(FeCoNi)	1D carbon nanofiber	B2	[35]
	Pd(FeCoNiCu)	2D mesoporous carbon nanosheets	L1_2_	[36]
	Pt(FeCoNiCu), Pt(FeCoNiZn), Pt(FeCoNiMn)	2D mesoporous carbon nanosheets	L1_0_	[37]
Oleylamine-Mediated Wet-Chemical Synthesis	Pd_0.10_Rh_0.10_lr_0.22_Pt_0.08_Sn_0.50_, Pd_0.09_Rh_0.09_lr_0.06_Pt_0.21_Sn_0.55_	/	B8_1_	[38]
	Pd_0.31_Rh_0.08_lr_0.05_Pt_0.07_Sn_0.50_, Pd_0.11_Rh_0.28_lr_0.06_Pt_0.08_Sn_0.48_	/	B8_2_	[38]
	(CoNiRhIrRu)Sb_3_	/	B8_1_	[39]
	(PtRh)(BiSnSb)	/	B35	[40]
	(NiPdPtRhIr)Zn, (NiFeCoPdPt)Zn, (NiFePdPtIr)Zn	/	D8_2_	[41]
	(NiPdPtRhIr)In, (NiFeCoPdPt)In, (NiFePdPtIr)In	/	B2	[41]
	(NiPdPtRhIr)Sn, (NiFeCoPdPt)Sn, (NiFePdPtIr)Sn	/	B8_1_	[41]
Other Methods	(PtPdAgRu)Cu, (PtPdAg)(CuFe)	/	L1_1_	[26]
	FeCoNiAlTi	/	L1_2_	[42]

**Table 2:** Comparation of synthesis strategies for HEI.

Synthesis Strategies	Advantage	Disadvantage
Carrier-Supported Annealing	• Good scalability for large-scale production • High reproducibility • Easy to obtain HEI phase	• Sensitive to support stability • High energy consumption
Space-Constrained Annealing	• Precise particle size control • Good HEI structural uniformity	• Complicated synthesis procedure • Limited scalability • High cost
Oleylamine-Mediated Wet-Chemical Synthesis	• Excellent compositional tunability • Wide application scenarios	• Limited scalability • Poor reproducibility • Additional ligand removal
Template-Based Epitaxial Growth	• High structural uniformity • Precise interfacial structural design	• Complicated synthesis procedure • Limited scalability • High cost
One-Step Chemical Dealloying	• Simple processing route • High scalability • Good reproducibility	• Limited composition control • Poor structural uniformity

**Table 3:** Catalytic performance of HEI for EOR.

Name	Composition	Synthesis Methods	Structure Features	Electrolyte	Mass Activity (A mg^−1^)	Reference
(PtRh)(BiSnSb)	Pt:Rh:Bi:Sn:Sb = 37.9:9.7:31.7:8.8:11.9	Oleylamine-mediated wet-chemical synthesis	B35	1 M KOH + 1 M C_2_H_5_OH	15.56	[40]
(PtRh)(FeNiCu)	Pt:Rh:Fe:Ni:Cu:= 1:3.23:0.95:0.84:0.89	Space-constrained annealing	L1_0_	0.1 M HClO_4_ + 0.2 M C_2_H_5_OH	0.914	[20]
(PtPdAu)(FeCoNiCuSn)	Pt:Pd:Au:Fe:Co:Ni:Cu:Sn= 0.8:0.1:0.1:0.6:0.1:0.1:0.1:0.1	Carrier-supported annealing	L1_0_	1 M KOH + 1 M C_2_H_5_OH	0.0125	[21]
(PtIr)(FeMoBi)	Pt:Ir:Fe:Mo:Bi= 1:1:0.22:1:0.46	Space-constrained annealing	L1_0_	1 M KOH + 1 M EG	5.2	[25]
Commercial Pt/C	/	/	/	1 M KOH + 1 M C_2_H_5_OH	1.50	[40]
PtBi/Pt	Pt:Bi=60.5:39.5.	Wet-chemical synthesis	core/shell	1 M KOH + 1 M C_2_H_5_OH	5.95	[124]
PtBi@PtRh_1_	Pt:Bi:Rh=49.3:47.1:3.6	Oleylamine-mediated wet-chemical synthesis	core/shell	1 M KOH + 1 M C_2_H_5_OH	6.87	[120]
Pd_61_Pt_22_Cu_17_	Pd:Pt:Cu=61:22:17	Wet-chemical synthesis	nanoring	1 M KOH + 1 M C_2_H_5_OH	12.42	[125]
PdZn/NC@ZnO	/	Hydrothermal synthesis	core/shell	1 M KOH + 1 M C_2_H_5_OH	18.14	[126]
Pt_47_Sn_12_Cu_41_	Pt:Sn:Cu:=47:12:41	Hydrothermal synthesis	nanoframe	0.5 M H_2_SO_4_ + 1 M C_2_H_5_OH	3.10	[127]
Pt-Rh	Pt:Rh=51.6:48.4	Oleylamine-mediated wet-chemical synthesis	nanowires	0.1 M HClO_4_ + 0.5 M C_2_H_5_OH	1.55	[128]
Pt_3_Fe	Pt:Fe= 88.4:11.6	Wet-chemical synthesis	nanowires	0.1 M HClO_4_ + 0.5 M C_2_H_5_OH	1.0	[129]

**Table 4:** Catalytic performance of HEI for ORR.

Name	Composition	Synthesis Methods	Structure Feature	Electrolyte	Half-Wave Potential (V)	Reference
Pt(FeCoNiCuZn)_3_	Pt:Fe:Co:Ni:Cu:Zn= 24.3:24:15:15.2:13:7.5	Carrier-supported annealing	L1_2_	0.1 M HClO_4_	0.922	[27]
(PtIr)(FeCoCu)	Pt:Ir:Fe:Co:Cu= 34.8:12.3:21.7:20:11.2	Carrier-supported annealing	L1_0_	0.1 M HClO_4_	0.894	[30]
Pt_4_FeCoCuNi	Pt:Fe:Co:Cu:Ni= 40.2:13.2:22:13.1:11.5	Carrier-supported annealing	L1_0_	0.1 M HClO_4_	0.943	[19,32]
(PtPd)(FeCoNi)	Pt:Pd:Fe:Co:Ni= 34.2:15.5:23.1:14.4:12.8	Space-constrained annealing	L1_0_	0.1 M KOH	0.910	[33]
PdFeCoNiCu	Pd:Fe:Co:Ni:Cu= 14:27:31:19:9	Space-constrained annealing	L1_2_	0.1 M KOH	0.900	[36]
Commercial Pt/C	/	/	/	0.1 M HClO_4_	0.861	[32]
Pd-Pt	Pt:Pd=75.6:24.4	Wet-chemical synthesis	nanodendrite	0.1 M HClO_4_	0.890	[145]
Pt_80_Fe_20_	Pt:Fe=4:1	Wet-chemical synthesis	nanowires	0.1 M HClO_4_	0.840	[146]
Pt-Ni	Pt:Ni=3:2	Wet-chemical synthesis	nanocages	0.1 M HClO_4_	0.915	[147]
Pd-Pt	/	Template-based epitaxial growth	nanosheets	0.1 M KOH	0.930	[148]
PtMn_3_N	Pt:Mn=50:121	KCl-matrix protection strategy	L1_2_	0.1 M KOH	0.919	[149]
AlFeCoNiCr	Al:Fe:Co:Ni:Cr= 97.5:0.5:0.5:0.5:0.5:0.5	Dealloying	fcc	0.1 M KOH	0.900	[150]

**Table 5:** Catalytic performance of HEI for HER.

Name	Composition	Synthesis Methods	Structure Feature	Electrolyte	Overpotential@10mA cm^−2^ (mV)	Reference
Pt_4_FeCoCuNi	Pt:Fe:Co:Cu:Ni = 40.2:13.2:22:13.1:11.5	Carrier-supported annealing	L1_0_	1 M KOH	20	[19,32]
(RuPt)(FeCoNi)	Pt:Ru:Fe:CoNi = 45:13:14:15:14	Space-constrained annealing	B2	1 M KOH	56 (@200 mA cm^−2^)	[35]
PtCuPdAgRu	Pt_:_Cu:Pd:Ag:Ru = 44.9:30.4:8.5:9.6:6	Template-based epitaxial growth	L1_1_	0.5 M H_2_SO_4_	24	[26]
FeCoNiAlTi	/	One-step chemical dealloying	L1_2_	1 M KOH	88	[42]
IrMo_0.59_	/	Wet-chemical synthesis	fcc	1 M KOH	38	[156]
IrCo	/	Carrier-supported annealing	hcp	1 M KOH	45	[157]
RuCo	Ru:Co = 94:6	Wet-chemical synthesis	nanosheets	1 M KOH	40	[158]
Pt_2_Ni_3_-P	Pt:Ni = 41.8:58.2	Wet-chemical synthesis	nanowires	1 M KOH	51	[159]
PtNi-O	Pt:Ni = 60.5:39.5	Carrier-supported annealing	heterogeneous	1 M KOH	79	[160]
Pt/MgO	/	Carrier-supported annealing	nanosheets	0.5 M H_2_SO_4_	39	[161]
Pt cluster/MoO_2_	/	Room-temperature light-reduction method	nanosheets	0.5 M H_2_SO_4_	33	[162]
PtRu/RFCS	Pt:Ru = 1:46	Wet-chemical synthesis	hcp	0.5 M H_2_SO_4_	46.7	[163]
Pt@MoS_2_/NiS_2_	/	Wet-chemical synthesis and annealing	heterogeneous	0.5 M H_2_SO_4_	34	[164]
PtCo@NCNT	Pt:Co = 2.9:1	Space-constrained annealing	hcp	0.5 M H_2_SO_4_	64	[165]

## Data Availability

Data sharing is not applicable to this article as no datasets were generated or analyzed during the current study.
